# MycoNews 2022: editorial, news, reports, awards, personalia, and book news

**DOI:** 10.1186/s43008-022-00106-1

**Published:** 2023-01-09

**Authors:** David L. Hawksworth

**Affiliations:** 1 Trait Diversity and Function, Royal Botanic Gardens, Kew, TW9 3DS Surrey UK; 2 Department of Life Sciences, The Natural History Museum, Cromwell Road, London, SW7 5BD UK; 3 Jilin Agricultural University, Changchun, 130118 Jilin Province China; 4 Geography and Environmental Science, University of Southampton, Highfield Campus, Southampton, SO17 1BJ UK

**Keywords:** Book reviews, International commission on the taxonomy of fungi, International mycological congress, Meeting reports, Obituaries, Tipping point

## Abstract

This fourth annual edition of *MycoNews* starts with an editorial asking if mycology is approaching a tipping point, and note of the journal’s 2021 Impact Factor almost doubling from 2020. Updated information and new speakers for IMC12 in 2024 is presented. Reports are provided for the Rise of the Fungi symposium in Amsterdam and of MycoRiseUP! in Warsaw in 2022. Information on activities of the International Commission on the Taxonomy of Fungi (ICTF) in the last two years are presented, including the formation of new Working Groups. Procedures for the nomination of IMA awards and for nomenclature proposals to be presented at IMC12 are provided. The Westerdijk Institute awards to Feng-Yan Bai and Marc Stadler are recorded, and Michael Wingfield and Geoffrey Kibby are congratulated on special awards they have received. Tributes are paid to the passing of two distinguished mycologists during the year, John Parmelee and John Pitt. Reviews of six mycological books published in 2021–22 are also provided.

## IS MYCOLOGY NEARING A TIPPING POINT?

**Fig. 1:**
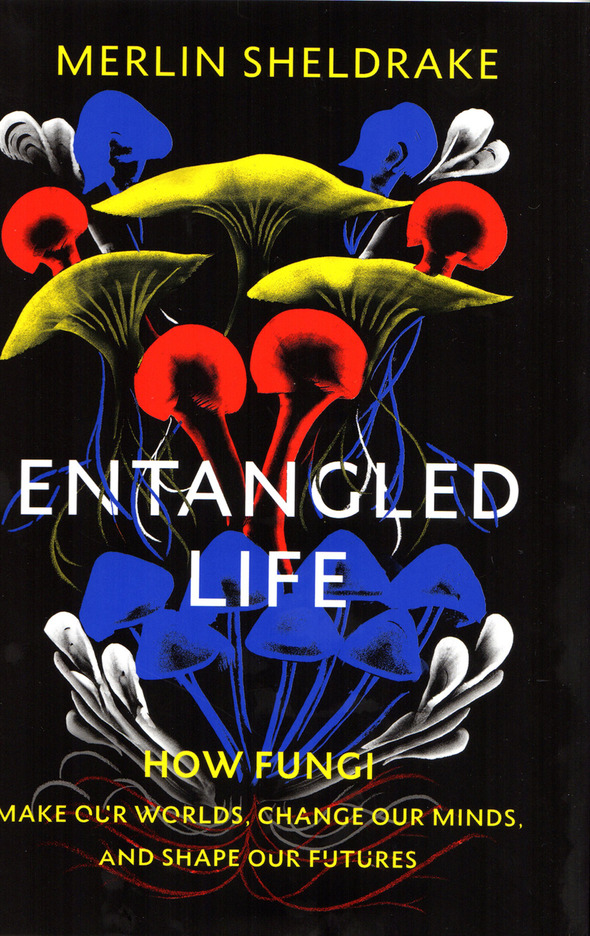
Entangled life (2020)

**Fig. 2:**
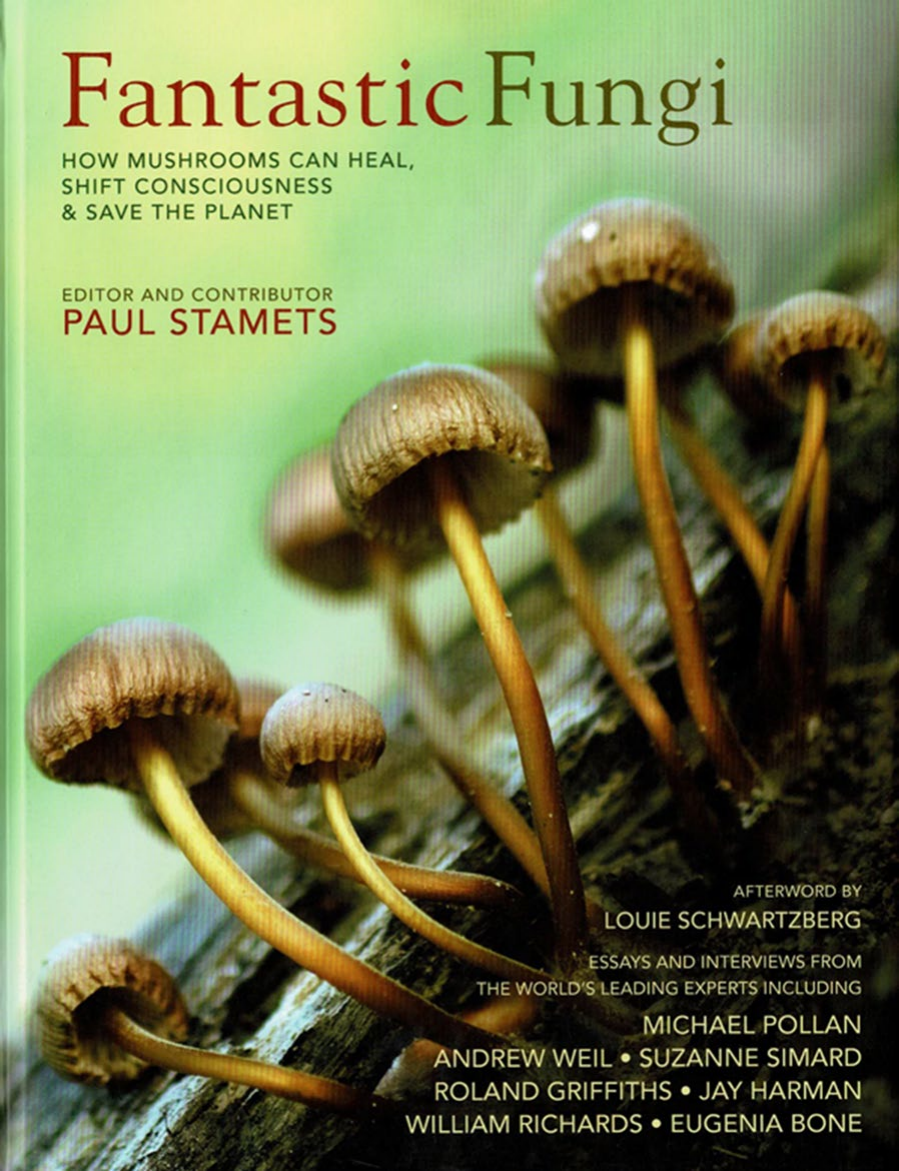
Fantastic fungi (2019)

On 29 November 2021, shortly after *MycoNews 2021* had gone to Press, it was announced that Melvin Sheldrake’s (2020) *Entangled Life: how fungi make our worlds, change our minds, and shape our futures* (Sheldrake 2020; Fig. 1)*,* was the 34th winner of the Royal Society of London’s annual Science Book Prize. The prize “celebrates the very best in popular science writing from around the world” and represents a tremendous achievement not only for Melvin personally but for mycology in general. The previous year the superbly illustrated *Fantastic Fungi: how mushrooms can heal, shift consciousness & save the planet* (Stamets 2019; Fig. 2) was made into a 1 h 20 film directed by Louie Schwartberg – *Fantastic Fungi: the mushroom movie* shown on NETFLIX, with trailers and enticing extracts on YouTube, and also available for purchase. As well as brilliant photography, this included interviews from diverse amateur and professional mycologists. The film captured public imagination and attracted a huge amount of attention; several people I knew with no previous interest in mycology suddenly became excited by the realization of what fungi are and the vital role they have in our lives and that of the planet. This was not the first film about mushrooms to appear in the decade and make TV, we also had *The Magic of Mushrooms* with Richard Fortey in 2014, and *The Kingdom of Fungi: how fungi made our world* with Lynne Boddy in 2018; both still available to watch online.

Since January 2018, at least 11 English-language books on fungi aimed at a general audience have been published, most of which have been received or review in *IMA Fungus* and included in the Book News sections. This compares with just two in the preceding five years of our Book News section (i.e. 2014–2017); this represents an increase in output of this genre by a staggering 550%! These numbers exclude text-books, regionally-oriented works, and ones directed at edible mushroom hunters or recreational-users – all categories which have also seen similar increases.

In the UK, we are also seeing more mentions of fungi in nature programmes of various kinds both on TV and radio channels. Concepts such as the “wood-wide web” are becoming really entrenched in the psyche of natural history presenters. And 2021 saw the recognition of IUCN (International Union for the Conservation of Nature) to use mycologically inclusive language in matters of species conservation add to refer to “flora, fauna and funga” in their internal and public communications, as reported in *MycoNews 2021*.

This dramatic rise in visibility of fungi to a wider public over the last decade has been remarkable in the history of mycology. It has led me to speculate that mycology is approaching a “tipping point”; the point at which a series of small changes or incidents becomes significant enough to cause a larger, more important change. There are numerous examples of this paradigm in the book by Gladwell (2000) which launched the concept. If we are indeed approaching a point where the importance of mycology will take-off and become established at a heightened level, from schools and universities, in local and national environmental and conservation actions, and in the public at large, we need to capitalize on the current awareness. This is something to which all mycologists can contribute, even for just a few hours each month; some suggestions for actions each of us can take have been made (Hawksworth 2003).

In the longer term, mycology deserves recognition as a “megascience” (Hawksworth 2009), something meriting a major international research infrastructure of its own. We are, however, unlikely to make more progress to that end if each of us assumes that action will be taken by *'somebody'*. If we have that mind-set, I fear we will find it is taken by *'nobody'*, and that we do not reach the tipping point that now appears to becoming a prospect. I was labelled a “fungal chauvinist” long ago by the late Lord May of Oxford (May 1991), but surely all of us who work with fungi should ensure we merit such a label.

**David L Hawksworth**

*Hon. President, IMA*

(d.hawksworth@kew.org)

## *IMA FUNGUS* IMPACT FACTOR ALMOUST DOUBLES

On 28 June 2022, Clarivate Analytics published the 2021 Journal Citation Report, which showed that *IMA Fungus* achieved a new 2-year Impact Factor (IF) of 8.044, and the 5-year Impact Factor of 6.756. The 2-year figure almost doubled from the 4.377 attained in 2020. The figures are based on the number of citations papers which appeared in the journal in the previous two years (i.e. 2019 and 2020) received in the following year (i.e. 2021). The journal was ranked fifth out of the 29 journals Clarivate place in the “mycology” category.

While the figure can be expected to fluctuate from year to year in journals such as *IMA Fungus* which publish a limited number of papers which will differ in the breadth of their readerships, this is the highest figure it has received since it was first allocated an IF for 2016 in 2017. The increase shows that papers in the journal are reaching a wide audience with many readers finding them of sufficient value to cite in their own publications.

If you have a contribution which you feel may appeal to wide range of mycologists, do consider *IMA Fungus* as a possible outlet for your research. In case you are uncertain your work will be considered as appropriate, the Editor-in-Chief will be pleased to receive initial enquiries by e-mail to avoid your having to submit through the electronic manuscript submission system and then being disappointed.

## IMC12: Fungal Biology and Applications – UPDATE

### For the very latest information on IMC12, please also go to www.imc12.org.

**Fig. 3:**
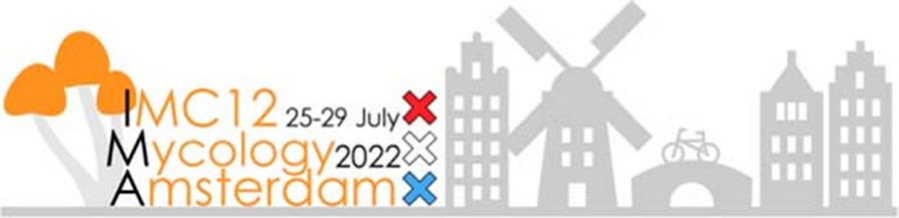
IMC12 logo

The 12th International Mycological Congress (IMC12; Fig. 3) will be held during 8–12 July 2024, in the MECC Conference Centre in Maastricht (in the South of The Netherlands). Maastricht is located in the heart of the Euregion, the area where The Netherlands, Germany and Belgium meet, home to four million people living in these three countries and where four different languages are spoken.

The MECC in Maastricht is also easily accessible by train via several major airports, or directly by plane to the Maastricht-Aachen Airport (10 km from MECC Maastricht and only 15 min away by car, taxi or bus). The Dutch Mycological Society, in collaboration with the Westerdijk Fungal Biodiversity Institute, is therefore pleased to invite you to attend the 12th International Mycological Congress in Maastricht. We are excited for the return of this incredible mycological experience to Europe, and to The Netherlands specifically. In January 2023, there will be a call for Symposia, and Workshops. We will keep you posted! The Scientific Themes have been established, and the chairs appointed. Each theme also has its own Scientific Committee.

*General Chair*: Pedro Crous.

*Scientific Chair*: Teun Boekhout.

**Theme 1: Cell biology, biochemistry and physiology**

Chair 1, Ida van der Klei, NL

Chair 2, Gregory Jedd, Singapore

Chair 3, Marcio Rodriques, Brazil

*Keynote lectures: Kaustuv Sanyal* (India)*, and Meritxell Riquelme* (Mexico)

**Theme 2: Environment, ecology and interactions**

Chair 1, Lynne Body, UK

Chair 2, Duur Aanen, NL

Chair 3, Yu Fukasawa, Japan

*Keynote lectures: Håvard Kauserud* (Norway)*, and Maiko Kagami* (Japan)

**Theme 3: Evolution, biodiversity and systematics**

Chair 1, Jos Houbraken, NL

Chair 2, Ester Gaya, UK

Chair 3, Lei Cai, China

*Keynote lectures: Toni Gabaldón* (Spain), *and Jolanta Maria Miadlikowska* (USA)

**Theme 4: Fungal pathogenesis and disease control**

Chair 1, Dee Carter, Australia

Chair 2, Martijn Rep, NL

Chair 3, Juan McEwen, Colombia

**Theme 5: Genomics, genetics and molecular biology**

Chair 1, Miia Mäkelä, Finland

Chair 2, Ronald de Vries, NL

Chair 3: Brenda Wingfield, South Africa

**Theme 6: Nomenclature**

Chair 1, Tom May, Australia

Chair 2, Konstanze Bensch, Germany

Chair 3, Bevan Weir, New Zealand

*Keynote lectures: M. Catherine Aime* (USA), *and Robert Lücking* (Germany)

**Theme 7: Applied mycology**

Chair 1, Lene Jesperson, Denmark

Chair 2, Richard Bélanger, Canada

Chair 3, Nancy Keller, USA

### Final keynotes announced!

Details of the keynote speakers who had already agreed to participate were included in MycoNews 2020 (*IMA Fungus* **11**(28): 4–5, 2020) and 2021 (**12**(36): 17–18, 2021) and are noted above. Each keynote session will have two speakers and the final ones can now be announced now:

### Fungal pathogenesis and disease control

*Eva Stukenbrock*, Germany

**Fig. 4:**
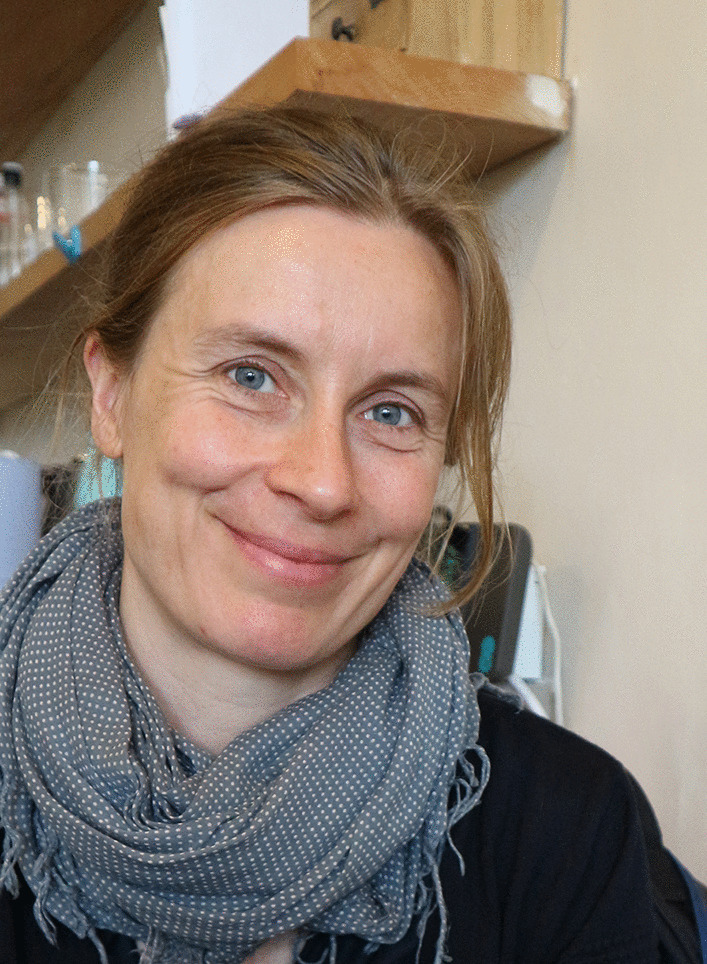
Eva Stukenbrock

The research of Eva Stukenbrock (Fig. 4) focuses on the ecological interactions and co-evolution of fungi associated with plants. Since her PhD at the ETH in Zurich, she has used the plant pathogenic fungus *Zymoseptoria tritici* as a model to study pathogen evolution. During her post doc at Aarhus University, Denmark she worked with Mikkel Schierup to apply whole genome coalescence analyses to infer the speciation history of *Z. tritici* and related *Zymoseptoria* species. In 2010, she was appointed group leader at the Max Planck Institute for Terrestrial Microbiology in Marburg, Germany, and since 2014 she is Max Planck Fellow and professor at Kiel University. Her group integrates computational biology with experimental and molecular approaches to study mechanisms of host specialization of plant pathogens.

*Judith Berman*, Israel

**Fig. 5:**
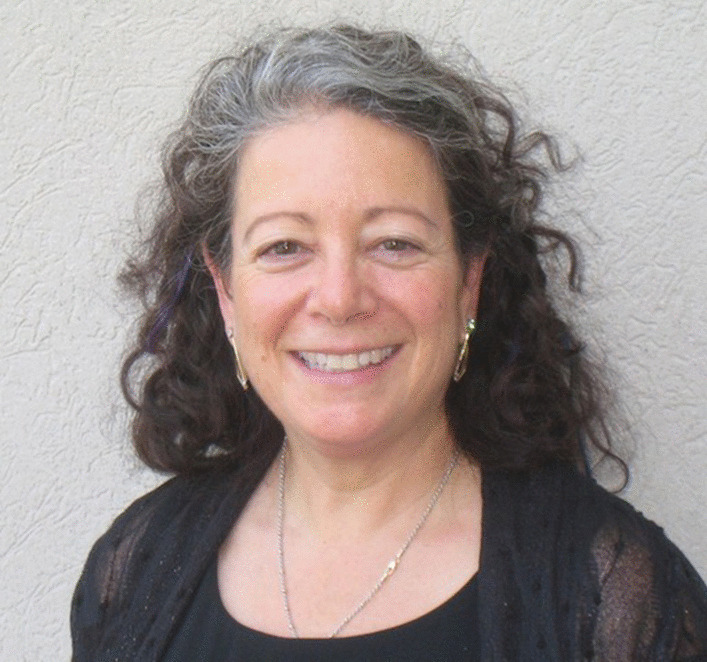
Judith Berman

Judith Berman (Fig. 5) is Professor and Nathan Galston Chair of Antimicrobial Drug Research at Tel Aviv University and Distinguished McKnight University Professor Emerita at the University of Minnesota. Her laboratory group studies basic mechanisms that underlie rapid phenotypic responses to stress, using yeasts, especially *Candida albicans* and *C. glabrata*. Her work is focused on genomic and epigenetic/metabolic responses to antifungal drugs, with a focus on ploidy shifts and antifungal tolerance. Her lab also studies the contributions of chromosome components including centromeres, telomeres, origins of replication and repeated DNA regions to genome stability and stress responsiveness. Berman and colleagues have developed and adapted many widely-used resources for the community of *Candida* researchers including the use of epitope and fluorescent protein tags and a range of tools for analysis and visualization of the genome structure of individual isolates. Judith did her PhD in yeast molecular biology with Ada Zamir at the Weizmann Institute of Science, Israel and as a post-doctoral fellow with Bik-kwoon Tye at Cornell University in Ithaca, NY. She has been a Professor at Tel Aviv University in Ramat Aviv, Israel since 2012 after 25 years at the University of Minnesota. She is an elected member of the European Molecular Biology Organization (EMBO), and an elected fellow of the American Academy of Microbiology (AAM) and the American Association for the Advancement of Science (AAAS).

### Genomics, genetics and molecular biology

*Hanna Johannesson*, Sweden

**Fig. 6:**
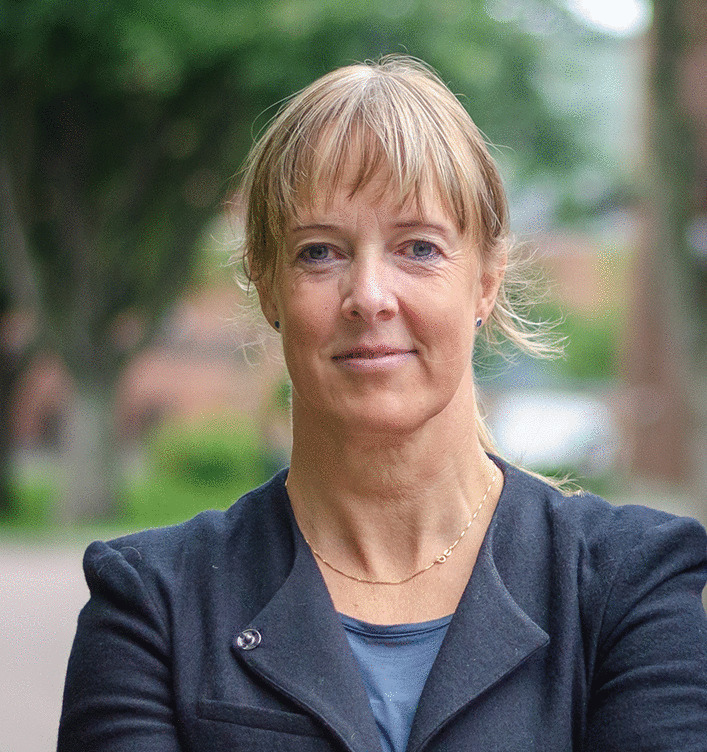
Hanna Johannesson

Hanna Johannesson (Fig. 6) is a Professor in Evolutionary Genetics at Uppsala University, Sweden. She established her own independent research group at the university in 2005, after a PhD at the Swedish Agricultural University and a postdoc at University of California at Berkeley. She achieved an Associate Professorship (docentur) in 2006 and between 2007 and 2013 she held a senior research position (rådsforskartjänst) funded by the Swedish Research Council (VR). Since December 2013 she has been a full professor. Hanna's research interest lies in the interface between mycology and evolutionary biology. Her group uses fungi as models to explore general evolutionary questions such as natural selection operating at multiple levels in the biological hierarchy, the causes and consequences of symbioses and switches in reproductive mode. In most of the work, she has a genomics approach. In particular, with funding from VR and the European Research Council (ERC) she was given the opportunity to dive deep into the causes and evolutionary consequences of meiotic drive: spore killing.

*Sakkie Pretorius*, Australia

**Fig. 7:**
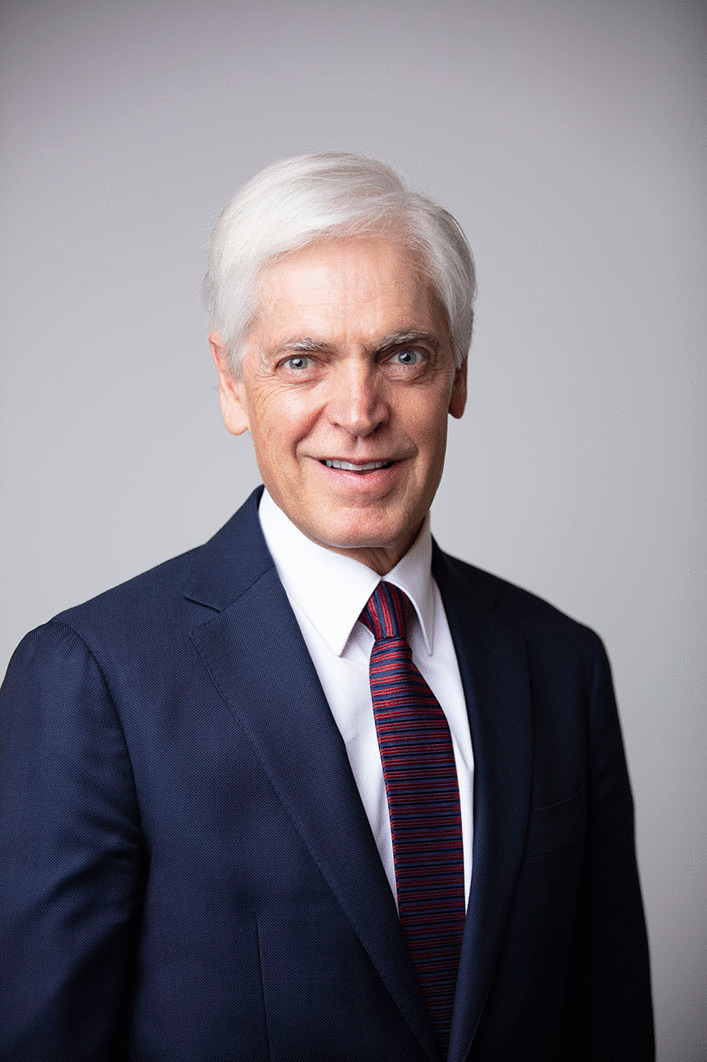
Sakkie Pretorius

Sakkie Pretorius (Fig. 7) is Deputy Vice-Chancellor: Research at Macquarie University in Sydney. He has a background in synthetic yeast genomics and wine biotechnology. Sakkie began his career in South Africa. At Stellenbosch University, he became the founding Director of South Africa’s Institute for Wine Biotechnology. In the US and Europe, he conducted research at the Albert Einstein College of Medicine in New York, the Max Planck Institute for Biophysical Chemistry in Göttingen, Germany, and the Katholieke Universiteit Leuven in Belgium. In 2003, Sakkie relocated to Adelaide to take up the role of Managing Director of the Australian Wine Research Institute. In 2013 he moved to his current role at Macquarie University where he also initiated a research programme in Synthetic Biology and founded the Australian Genome Foundry. He is the driving force behind a university-wide BioInnovation Strategy and leads the Australian team of the *International Synthetic Yeast Genome* project. Sakkie has published 230 research papers, delivered 600 conference presentations, won research grants totalling more than $120 million, achieved many awards, and filed six patents. Over the past three decades, he has supervised or co-supervised 33 PhD students and 56 MSc students.

### Applied mycology

*Martin Kimanya*, Tanzania

**Fig. 8:**
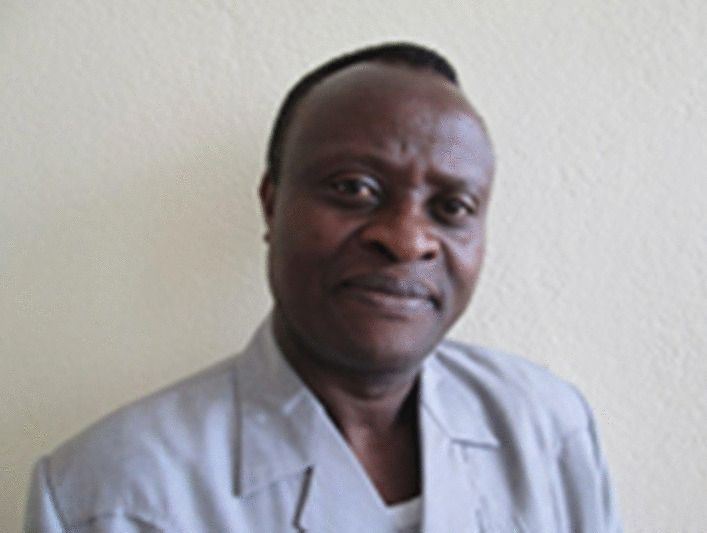
Martin Kimanya

Martin Epafras Kimanya (Fig. 8) is an associate Professor of Food and Nutrition Sciences at the Nelson Mandela African Institution of Science and Technology (NM-AIST). He is a holder of PhD in Applied Biological Sciences and Master’s and Bachelor’s degrees in Food Science and Technology. While keeping his role as a supervisor of PhD candidates and an active participant in research projects at NM-AIST, Martin is currently serving as the Sanitary and Phytosanitary (SPS) Technical Expert for the East African Community Market Access Upgrade Programme (MARKUP), funded by the European Union and the Government of Germany. Prior to joining MARKUP, Martin worked as Technical Advisor on Aflatoxin Control for the African Union Commission (January 2017–September 2018. For over 15 years, Martin has been participating in research on food safety and nutrition sciences with special interest in impact of aflatoxin and fumonisin exposure in humans. He has supervised several post-graduate students including 10 at PhD level. Martin is a recipient of several awards including appointment as the permanent corresponding member for the Belgium Royal Academy for Overseas Sciences, in the Section of Natural and Medical Sciences. He is a member of several scientific committees and advisory boards in Tanzania and abroad and has co-authored several peer-reviewed publications and book chapters.

*Matteo Lorito*, Italy

**Fig. 9:**
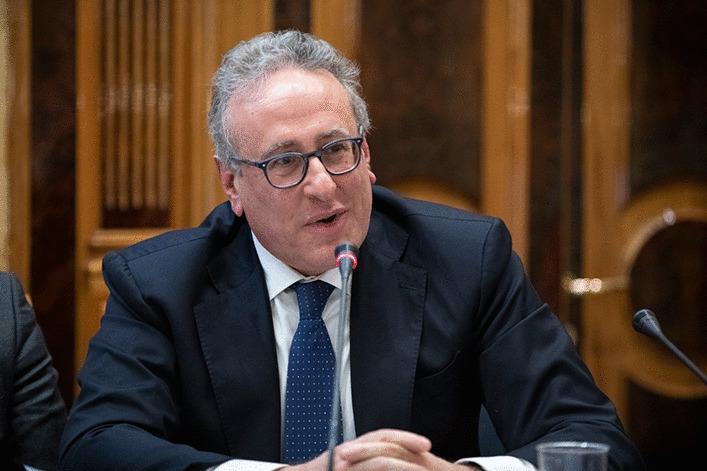
Matteo Lorito

Matteo Lorito (Fig. 9) is Rector of the University of Naples Federico II. He is Full Professor of Plant Pathology and Phytopathological Biotechnology and has been Director of the Department of Agricultural Sciences. He is the President of the Italian Society of Plant Pathology. His scientific activity has focused on the study of plant-environment micro/macroorganism interactions, from basic genetic/biotechnological research to field application, aimed at increasing the quality, quantity and sustainability of agricultural production. He is co-founder of three spin-offs/startups: Phytobials, Biomarinex and RE&DIT. He has contributed to the development of about 10 formulations on the global market (90 countries) applied as biopesticides and biofertilizers in collaboration with several multinational companies. He is an inventor in about 15 international patents and has collected funding on competitive calls for over 15 million euros. He has received many awards, including being elected a Fellow of the American Phytopathological Society, member of the European Academy of Science and Research, the International Prize "Guido Dorso" for having supported development and progress of Southern Italy, the Capo d'Orlando Scientific Award for the Science and Food section, and the Voce di Napoli Award - Forming the Future 2021 50 top Italy.

**Pedro W Crous**

(p.crous@wi.knaw.nl)

## FUNGAL NOMENCLATURE PROPOSALS – DEADLINE APPROACHING

Mycologists wishing to make proposals to amend the formal rules relating to the naming of fungi, that is *Chapter F* of the *International Code of Nomenclature for algae, fungi, and plants* (May et al*.* 2019) at IMC12 in 2024, are reminded that the deadline for submission of proposals is now **31 December 2023.** See May (2020) for details of how to prepare and submit proposals.

## FUNGAL EVOLUTION – upcoming meeting

**Fig. 10:**
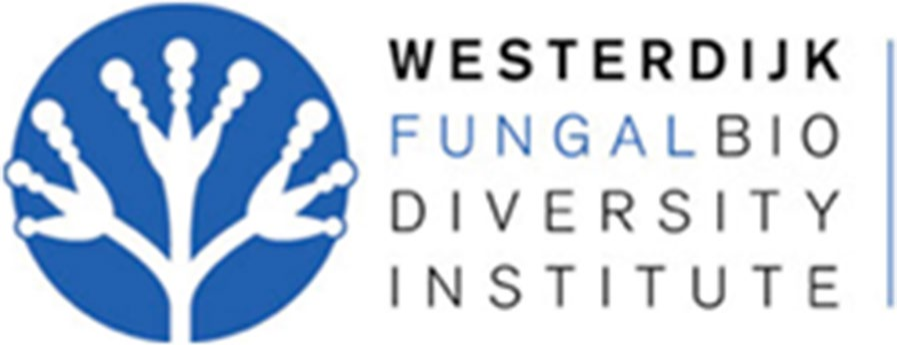
Westerdijk Fungal Biodiversity Institute logo

Following the success of the “Rise of the Fungi” meeting in 2022 (*see below*), the tradition of annual stimulating and focussed mycological symposia organized by the Westerdijk Fungal Biodiversity Institute (Fig. 10) is scheduled to continue in 2023 with one on the topic of “Fungal Evolution”. This will be held on 17–18 April 2023 at the KNAW headquarters, Trippenhuis, Amsterdam. This will also provide an opportunity for mycologists to discuss controversial proposals for possible changes in the rules governing the naming of fungi. Watch the Institute’s website (https://wi.knaw.nl/news/category/Meetings) for further information as the programme develops.

## REPORTS

### International commission on the taxonomy of fungi (ICTF) – actions 2020–2022

The International Commission on the Taxonomy of Fungi (ICTF) has seen a busy period over the last 2 years. This included producing publications with recommendations and discussions on publishing new species and names (Aime et al. 2021), sequence-based classification (Lücking et al. 2020; Lücking et al. 2021), and the use of italics for scientific names at all ranks (Thines et al. 2020). ICTF members have also been active in several additional projects (albeit without explicit ICTF involvement) related to classification and selecting names for phytopathogens (Crous et al. 2021), rusts (Aime & McTaggart 2021) and yeasts (Yurkov et al. 2021) to name a few. To provide a continued forum for discussion of these topics the ICTF implemented important administrative changes in 2021. Chiefly amongst these were to limit active membership to 20 and institute an Advisory Board from active ex-members. This is anticipated to allow for continuity, whilst expanding representation and diversity. The Board is expected to serve as a source for advice on new members and projects. Additionally, Andy Miller stepped down after valued service as Executive Vice-President and Marco Thines was selected as his replacement. All current members as well as the Board are listed on the ICTF website.

Based on internal discussions, eight new task and finish working groups were proposed for ICTF members, and conveners selected. In some cases, non-ICTF members with particular interest in a topic were invited to collaborate. The research focused topics are:

1. General purpose classification
2. Lists of protected family and generic names
3. Typification of older names
4. Naming environmental sequences
5. Revising rules regarding living cultures as types
6. Data standards for genomes

The additional two working groups are focused on transitioning the current ICTF website and exploring use of other media (Working Group 7) and revising the current ICTF statutes (Working Group 8) in order to further clarify executive roles, voting procedures, and the new Advisory Board. These topics will be reviewed over time and reassessed.

The goals of the ICTF remain to promote the science of fungal taxonomy, wherever it is practiced, and serve as a forum for discussion between mycologists at all stages of career development, whilst reflecting the diversity of our discipline. We hope to continue working towards this with all our colleagues and welcome any inquiries regarding our current activities.

**Conrad Schoch, Cathie Aime & Marco Thines**

(schoch2@ncbi.nlm.nih.gov)

### Westerdijk Spring Symposium: Rise of the Fungi

**Fig. 11:**
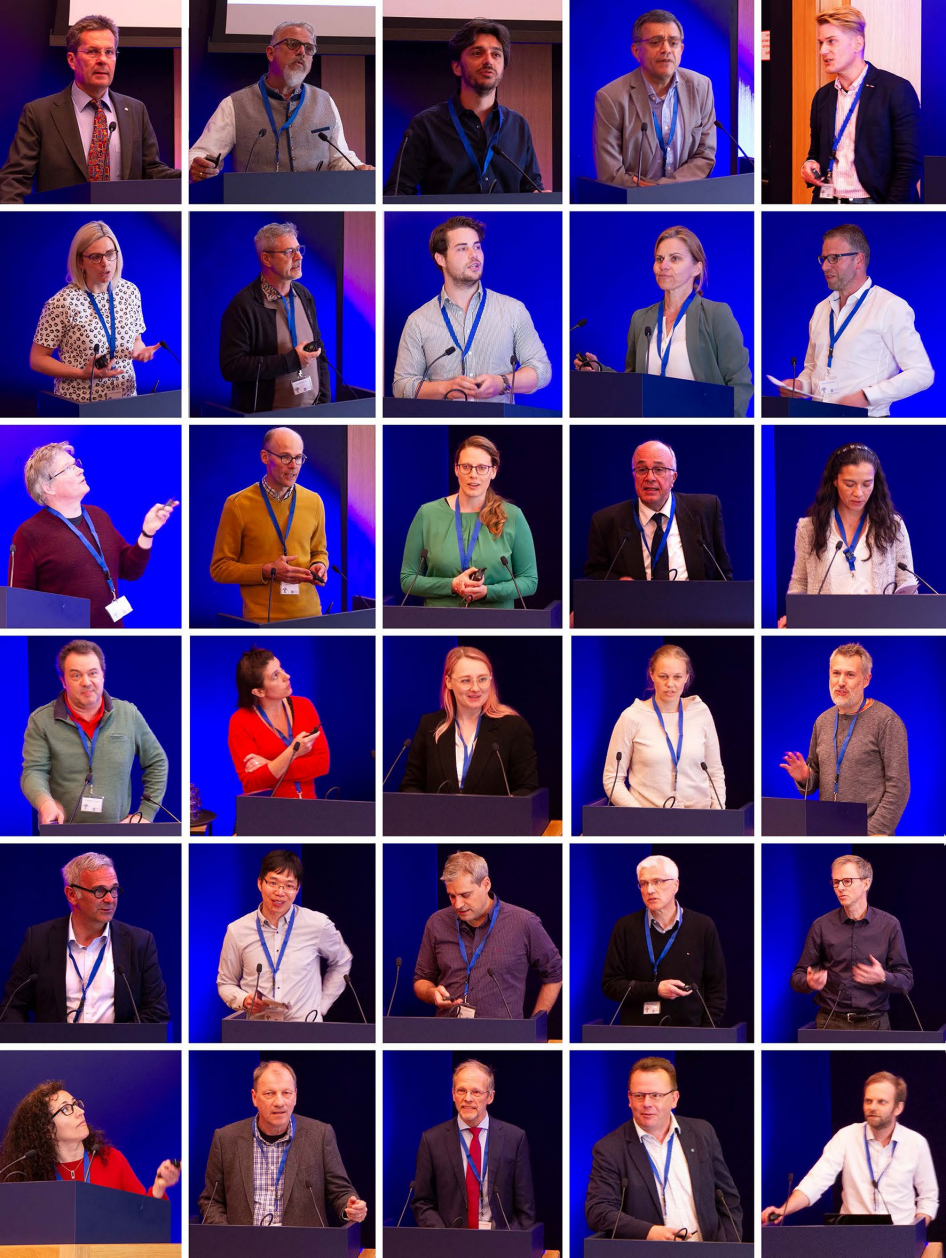
Rise of the fungi 2022 speakers

**Fig. 12:**
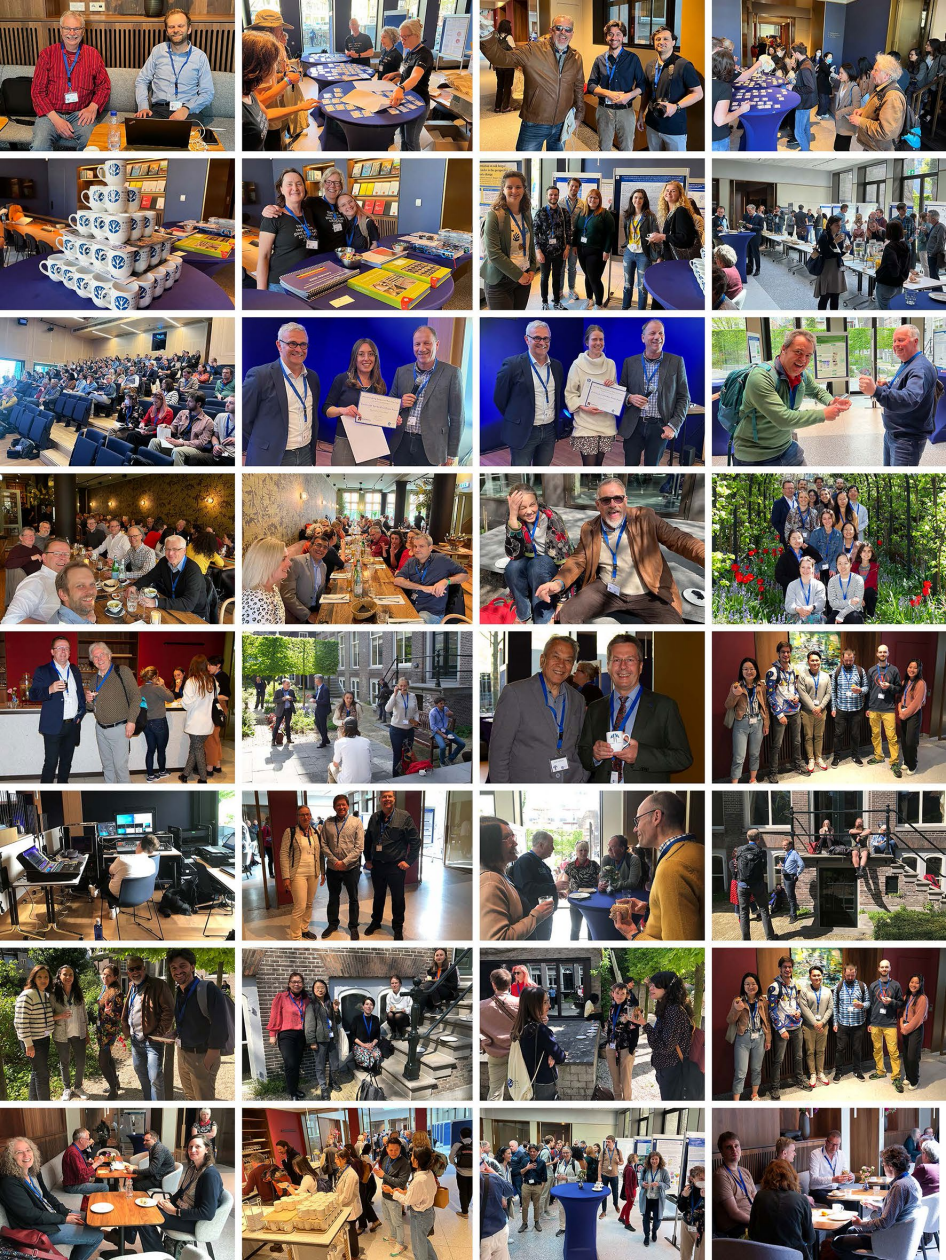
Rise of the fungi 2022 participants interacting outside the sessions

For 2022 the theme of the Westerdijk symposium was “Rise of the Fungi” (21–22 April 2022), which was held at Trippenhuis, the headquarters of the Royal Dutch Academy of Arts and Sciences in Amsterdam. The meeting had originally been planned for 2020 (hence that date appears on the commemorative mugs) but had to be postponed due to the Covod-19 restrictions in place. It was attended by 136 participants from 17 nationalities (excluding students, of whom several arrived on the day), a selection of whom are shown in Figs. 11 and 12.

On Thursday morning, the first session “Plant Health: fungi and climate change” (Matteo Garbelotto, Vladimiro Guarnaccia, Peter Bonants, Marco Thines) focussed on forest pandemics, fungal diagnostics, and the effect of climate change. The second session “Human Health: fungi on the move” (Vishnu Chaturvedi, Johanna Rhodes, Wieland Meyer, Auke de Jong) focussed on the emergence of fungal resistance, and molecular epidemiology. After a light lunch, the third session “Fungi and Food Security” (Sarah de Saeger, Markus Schmidt-Heydt, Simon Avery, Jan Dijksterhuis) took a detailed look at the global impact of fungal food spoilage and mycotoxins followed by the final session of the day, “Naming Fungal Taxa: from Linnaeus to Genomes”, where Gianluigi Cardinali presented his challenging views on taxonomy, followed by Robert Lücking who addressed a formal solution to naming “dark matter fungi”. The session was rounded off by a panel discussion on the topic, drinks, further discussions, poster awards, and a speaker’s dinner at a much favoured nearby Indonesian restaurant.

Friday started with the announcement of the Johanna Westerdijk Award made to Feng-Yan Bai, and the Josef Adolf von Arx Award to Marc Stadler (*see below*). The first scientific symposium on “Fungal Applications” (Axel Brakhage, Soizic Prado, Kristina Haslinger, Thomas Ostenfeld Larsen) focussed on genome mining, and natural products, followed by “Fungal Genomes and Taxonomy” (Toni Gabaldón, Like Fokkens, Mikael Andersen, Ronald de Vries), who addresses hybridisation, genomes and taxonomy. The first session after lunch focussed on “Fungal Evolution and Ecology” (Ester Gaya, Benedetta Turchetti, Aída Vasco-Palacios, Bart Theelen), and Teun Boekhout, who was a special speaker, reflecting on his past 40 years at the Westerdijk Institute, and future prospects for those starting to work on yeast taxonomy. The final session of the day addressed “Nagoya and DSI”, in which Jörg Overmann and Martin Brink discussed the implications of the Nagoya protocol for taxonomic research, and how access to and benefit sharing from Digital Sequence Information would impact future research. The symposium was concluded with a discussion and drinks, with everyone returning home extremely pleased at having been able to attend this mycological symposium so soon after the Covid-19 pandemic.

**Pedro W Crous**

(p.crous@wi.knaw.nl)

### MycoRise UP! 2022

**Fig. 13:**
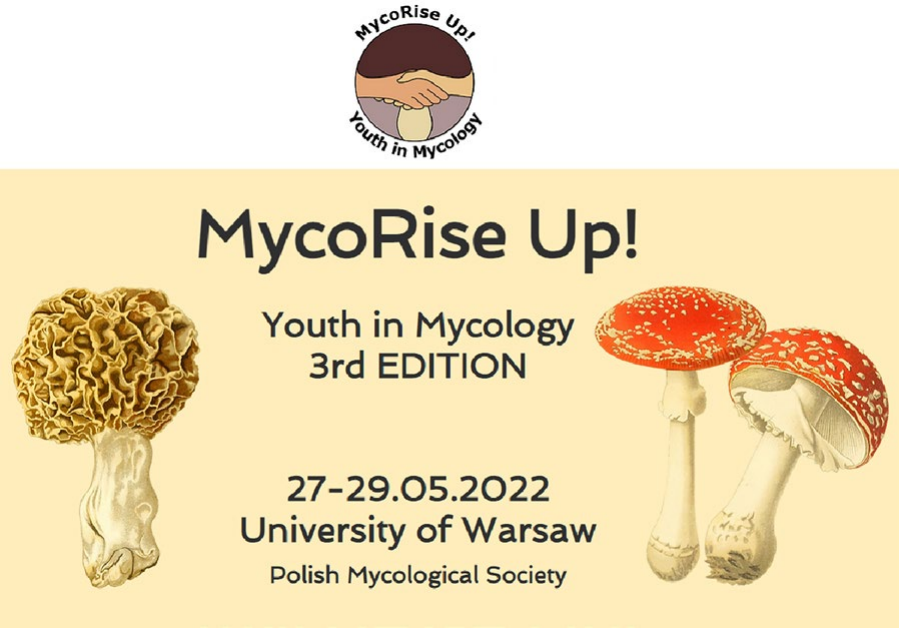
MycoRiseUP! 2022 logo

The MycoRise Up! (Fig. 13) meeting of young mycologists was held in Poland for the third time on 27–29 May 2022 in Warsaw, and for the first time as an international event. The Polish Mycological Society, Faculty of Biology and the Botanic Garden, University of Warsaw, and Institute of Biochemistry and Biophysics, Polish Academy of Sciences were the co-organizing institutions. The meeting itself was organized by mycology students: Beniamin Abramczyk, Kamil Kisło, Alicja Okrasińska, Małgorzata Orłowska, Grzegorz Ostrowski, and Igor Siedlecki from Warsaw, who were supported by a Scientific Committee, which consisted of researchers from the Czech Republic, Poland, and Macedonia.

**Fig. 14:**
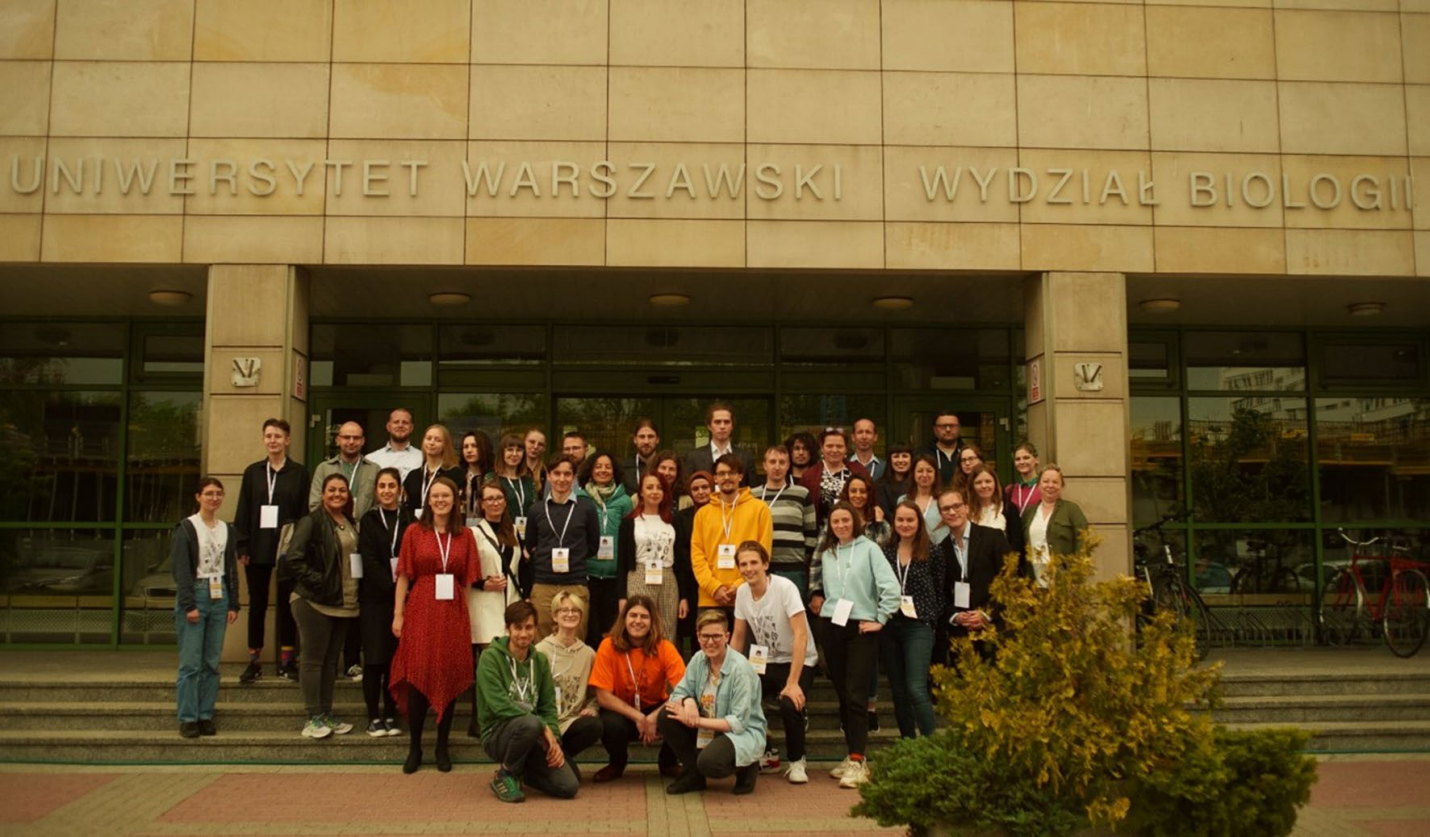
MycoRiseUP! 2022 group picture taken during the conference. *Photo*: Karolina Gołębiowska

The conference attracted 45 students from Poland, the Czech Republic, Macedonia, Germany, Iran, and China, representing 21 institutions (Fig. 14). The opening lecture entitled "How light affects fungal metabolism" was delivered by Grzegorz Janusz (Maria Curie-Skłodowska University, Lublin, Poland). Three plenary talks were given by members of the Scientific Committee: Ondřej Koukol (Charles University, Prague, Czech Republic), Katerina Rusevska (Ss. Cyril and Methodius University, Skopje, Macedonia), and Monika Urbaniak (Institute of Plant Genetics, Polish Academy of Sciences, Poznań, Poland). An additional lecture given by Julia Pawłowska (University of Warsaw, Warsaw, Poland) on GBIF and data handling standards was also organized on Saturday. The 24 students’ presentations and 21 posters were presented in five thematic sessions: Biology and Ecology of Fungi, Biotechnology of Fungi, Fungi in Interactions, Pathogenic Fungi, and Humans & Fungi. The prizes for the best presentations and posters were awarded to 11 participants. Students also had a chance to get to know each other better in an informal atmosphere during a party organized in the Botanic Garden of the University of Warsaw. On the last day of the conference, students could also participate in a mycological bioblitz in Bielany Forest (https://www.inaturalist.org/projects/mycoriseup2022).

The book of abstracts and information on the previous two conference are available at https://mycoriseup.wixsite.com/konferencja. During the meeting, it was agreed that the next MycoRise Up! in 2023 would be organized in Lublin (Poland), and we look forward to seeing you there!

**Beniamin Abramczyk, Kamil Kisło, Alicja Okrasińska, Małgorzata Orłowska,**

**Grzegorz Ostrowski, Igor Siedlecki & Julia Pawłowska**

(julia.z.pawlowska@uw.edu.pl)**AWARDS**

### IMA awards 2024

To acknowledge the various contributions of its members, the IMA is giving three awards in the following categories:

- **De Bary Medal - **Based on outstanding career research.
- **Ainsworth Medal - **For recognition of extraordinary service to world mycology.
- **Young Mycologist Award - **Awarded to outstanding mycologists early in their career.

The IMA Awards Committee for 2024 consists of the Council Members, and up to three members from the Executive Committee (EC). Chiharu Nakashima, head of the IMA Awards committee, will accept nominations for the Ainsworth Medal and the De Bary Medal. The nominations for the Young Mycologist Awards should be addressed to the Regional Mycological Member Organizations (RMMOs) of the region in which the Nominee achieved his research results. Contact details for the RMMOs are available on he IMA website.

The deadline for application will be on **30 January 2024**, to coincide with the IMC 12 meeting in Maastricht, The Netherlands the same year.

Requirements for application of the awards are the following:

- An individual may receive the same IMA Award only once.
- Self-nomination is not allowed.
- Nominators must be members of the IMA.
- Nominees who are not chosen for the prize may be re-nominated for up to two additional terms (within the year limit linked to the specific award).

Documents to be submitted should include a nominating letter, including a detailed evaluation of the nominee’s contributions to mycology, and a current *curriculum vitae*.

The awards consist of a certificate. Note that the Committee may not grant an award in a given year if there is no suitable candidate that meets the criteria. Presentation of the awards will take place at the awards ceremony at IMC 12 in 2024.

### WI-KNAW Fungal Biodiversity Institute Awards 2022

On the second day of the “Rise of the Fungi” symposium in Amsterdam on Friday 22 April 2022 (*see above*), the WI-KNAW Fungal Biodiversity Institute presented its two prestigious awards. The awards are made at irregular intervals by the institute following discussions by its senior staff. This is the ninth time these awards have been made, and the citations were read, and the presentation of certificates made, by the Institute’s Director, Pedro W. Crous.

### Johanna Westerdijk award: Feng-Yan Bai

*Awarded on special occasions to an individual who has made an outstanding contribution to the culture collection of the CBS Fungal Biodiversity Centre, marking a distinguished career in mycology. Nominees for the award will be evaluated on the basis of quality, originality, and quantity of their contributions to the collection, and on the basis of associated mycological research in general.*

**Fig. 15:**
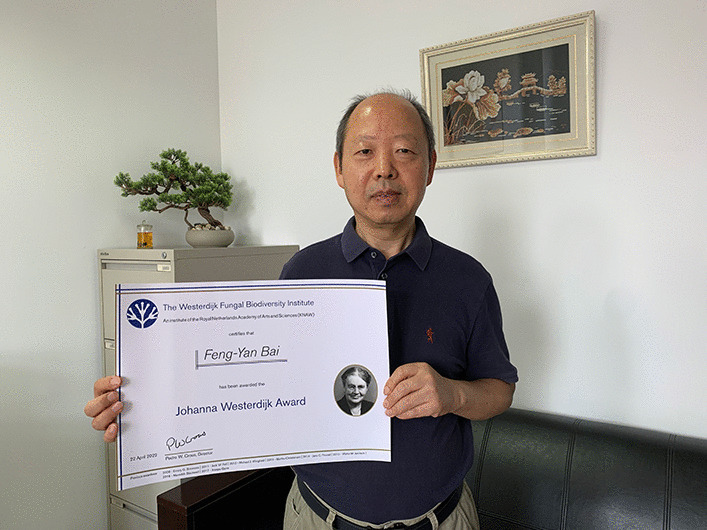
Feng-Yan Bai

Feng-Yan (Fig. 15) is one of the World’s leading authorities in mycology with particular expertise on the systematics, biodiversity, and evolution of yeasts. Throughout his career, he has had a monumental and tangible impact on mycology in Asia and globally. He is a sought-after speaker and organizer and continues to inspire the next generation of mycologists. His research has updated the overall taxonomic framework of yeast, as well as clarified the Far-East Asian origin of the lager beer yeast and the domesticated population of *Saccharomyces cerevisiae.* He has also had many other academic successes, awards and recommendations. He is recognized as a special recipient of the Westerdijk Award today, however, as he has deposited a huge collection of yeasts in the culture collection, thereby ensuring that these fungi remain available for research by future generations. These cultures were collected by him and his team over many years, and as such represent a major investment of time and resources. As mycological community, we thus thank him for this incredible foundation, and trust that students in years to come will continue to build on this wonderful platform.

### Josef Adolf von Arx Award: Marc Stadler

*Awarded on special occasions to an individual who has made an outstanding contribution to taxonomic research of fungal biodiversity, marking a distinguished career in mycology. Nominees for the award will be evaluated on the basis of quality, originality, and quantity of their contributions in the field of fungal taxonomy.*

**Fig. 16:**
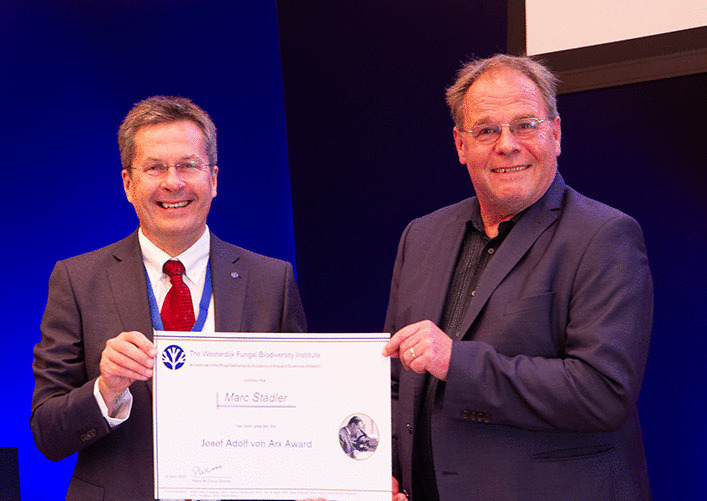
Marc Stadler

Marc Stadler (Fig. 16) obtained his PhD in Kaiserslautern in 1993. After a few years as a postdoc in Natural Product Chemistry in Sweden, he accepted a position at Bayer in 1995, and was appointed as Head of their Natural Product Chemistry/Microbiology Laboratory. From 2006–2012 he spent time at InterMed Discovery, Dortmund, Germany, and in 2012 became Head of the Department of Microbial Drugs, HZI Braunschweig. Marc serves on numerous committees and associations, and is well-known in mycological circles in Asia, Africa, Europe, Latin and North America, and has also been tasked with responsibility for international relations in the IMA. He is a well-known keynote speaker at major events, and is presently President-elect of the IMA. Marc has invested much time in supporting mycological journals in general, and as editor-in-chief of *Mycological Progress* in particular.

During his career, he has published a great number of papers, but most important of all, he has contributed greatly to method development for the cultivation of rare secondary metabolite producing microbes and fungi. Over the years he has been very successful in the discovery of novel bioactive metabolites of fungi, and the scale-up of fermentation and downstream processes. The contribution of Marc’s career to mycology is not seen only in terms of numbers and citations, but especially in terms of quality, relevance, and application. It thus gives us great pleasure to award Marc Stadler the Josef Adolf von Arx award for his fungal systematic research.

### Michael J. Wingfield

**Fig. 17:**
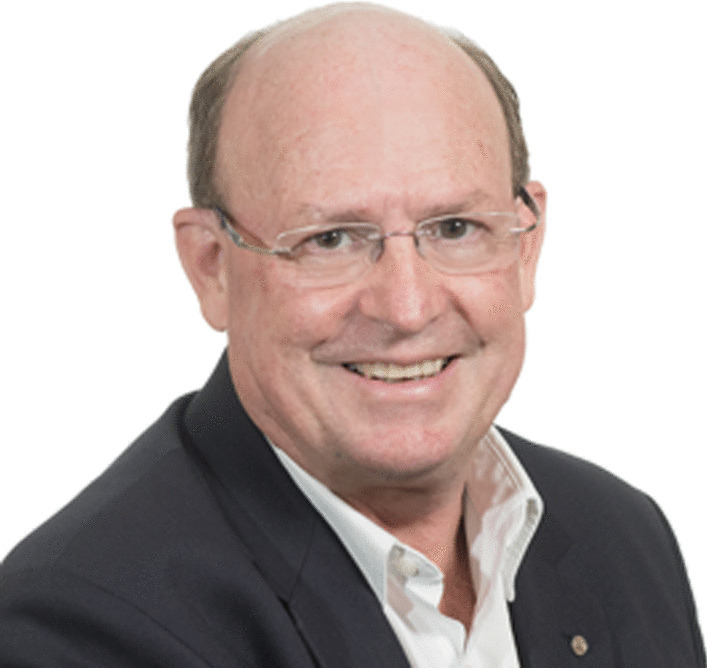
Michael J Wingfield

The IMA wishes to congratulate one of its most ardent supporters over many years, Mike Wingfield (Fig. 17), a professor at the University of Pretoria’s (UP) Forestry and Agricultural Biotechnology Institute (FABI), on his receipt of the annual Harry Oppenheimer Fellowship Award for his research into disease-causing fungi.

This R2 million award recognises scholarship of the highest calibre across various academic and research disciplines, and is ranked as one of Africa’s most prestigious research grants. The award was initiated by the Oppenheimer Memorial Trust in 2000 to commemorate Harry Oppenheimer, and his efforts to support human and intellectual development and to advance scholarship. The title of his project, “Quest to unravel the origin and ecology of two human pathogenic fungi and expand the base of medical mycology in South Africa”, will focus on fungi that cause diseases in humans in South Africa and elsewhere in the world. It aims to learn more about where disease-causing fungi live naturally, understand them more, and how to avoid the diseases they cause. In his acceptance speech he stressed that he saw his role primarily as one of mentorship: to pass on his knowledge and experience to younger scientists, and to share his passion for fungi and for research with others that might build on what he has been privileged to do.

### Geoffrey Kibby

**Fig. 18:**
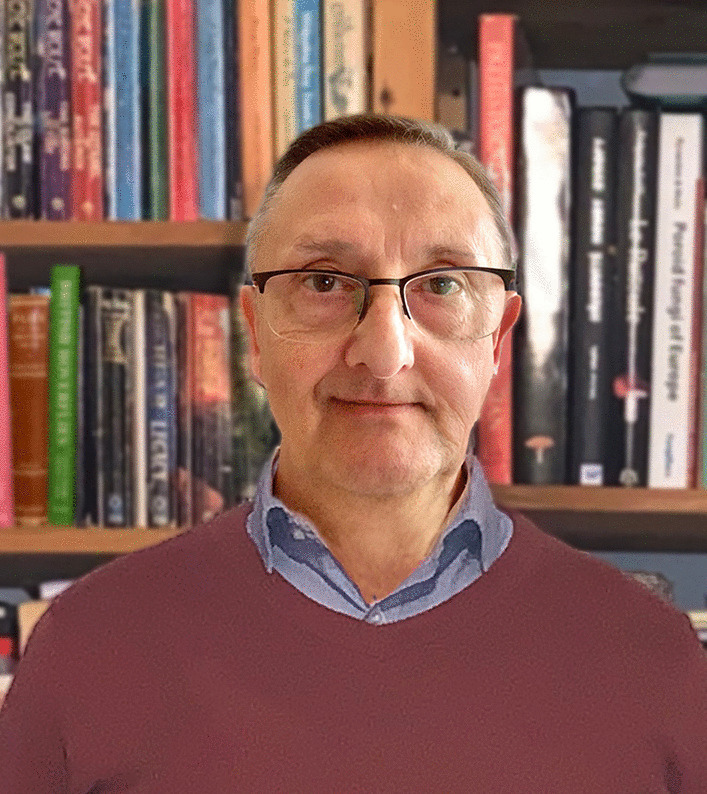
Geoffrey Kibby

The Linnean Society of London H. H. Bloomer Award for 2022 was presented to Geoffrey Kibby (Fig. 18) in the rooms of the Society at its Anniversary Meeting on 24 May 2022. One pf these awards is made each year for “an amateur naturalist for an important contribution to the knowledge of natural history”. This is the first time a mycologist has received this award since that presented to lichenologist and botanist Ursula K. Duncan (1920–1985) in 1973.

Although employed as an entomologist for many years, and yet with no formal mycological training, Geoffrey was always really a mycologist at heart and has been exceptionally productive in promoting field mycology and publishing treatments of different mushroom groups. Almost entirely self-taught, enhanced by contacts with mycologists at Kew and overseas, he has become a leading authority on a variety of mushroom genera publishing guides for their identification and taxonomic revisions of ones especially difficult to identify – invariably illustrated by his own superb coloured drawings, and updating them as new data become available. He is the editor and founder of the quarterly *Field Mycology*, published by the British Mycological Society since 2000, which is produced to the highest academic standards, superbly illustrated in full colour, and includes identification keys, taxonomic, and related articles, for example on new and interesting records and techniques. He is now embarked on a multi-volume self-illustrated authoritative series *Mushrooms and Toadstools of Britain and Europe*; three volumes have already appeared (Kibby 2017, 2020, 2021).

Geoffrey has become a much respected authoritative and willing source of support for the burgeoning field mycological community in the UK, and the important role he has grown into in the UK mycological scene has been recognized by his appointment as a Research Associate at the Royal Botanic Gardens Kew where he has some facilities and ready access to their internationally unrivalled fungal collections and comprehensive library facilities.

The IMA congratulates Geoffrey on this most well-deserved award and wishes him all success in his ongoing projects.

## IN MEMORIAM

### John [“Jack”] Parmelee (1924–2022)

**Fig. 19:**
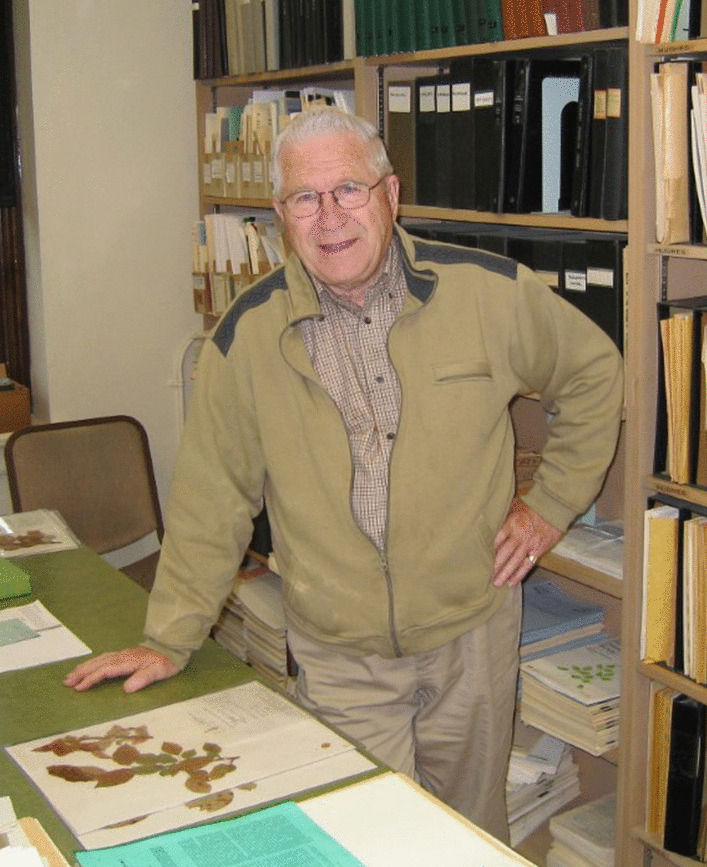
John Parmelee (1924–2022) in the William Saunders Building, Central Experimental Farm, Ottawa, June 2008. *Photo*: Scott A Redhead

Jack (Fig. 19) will be familiar to many mycologists around the world through his role as curator of the National Mycological Collection in Ottawa until his retirement in 1987, though he continued as an Honorary Research Associate and was still to be found there from time to time until he reached 90 in 2014.

He was raised in Ottawa and studied plant pathology at McDonald College (McGill), after which he started work at the Department of Agriculture in Ottawa in 1949 – but the next year moved to Toronto to pursue a Masters degree in mycology under H.S. Jackson. In 1961 he interrupted his career at the Department of Agriculture again and returned to Toronto to earn a PhD under the supervision of Roy F. Cain. He was the able to return as a Research Scientist at Agriculture and Agri-Food Canada. He conducted valuable work on obligate parasitic fungi, especially *Gymnosporangium,* making important contributions to agriculture in Canada and throughout the world through many research publications. He enjoyed fieldwork and travelled in the High Arctic and its islands as well as many of the country’s National Parks. He has left a legacy of valuable agricultural research and shared knowledge which has contributed much to the advancement of research on plant parasitic fungi in the Canada he loved so much.

A humble man with a quiet and constant integrity, Jack made life-long friends wherever he went, was the Master of Ceremonies at staff Christmas parties, and had a seemingly inexhaustible supply of anecdotes. He leaves many happy memories behind and all his colleagues will miss him.

### John Ingram Pitt (1937–2022)

**Fig. 20:**
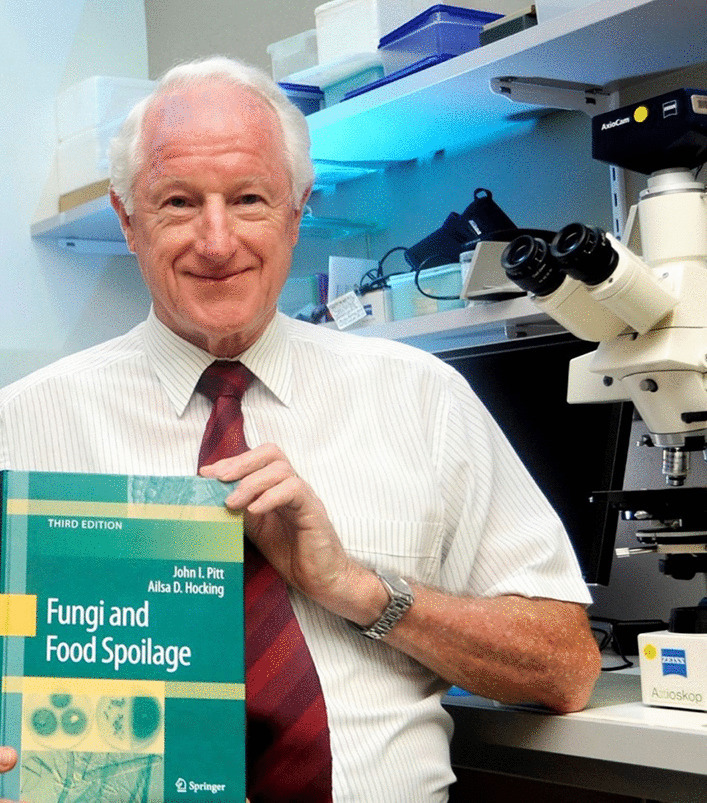
John I Pitt (1937–2022) with the third edition of fungi and food spoilage (2009)

It is with deep sadness we report the passing of John Pitt (Fig. 20) on 23 March 2022, after a long and courageous battle against lymphoma.

On 1 March 1954, just before his 17th birthday, John started his first job, joining the CSIRO Division of Food Preservation as a Technical Assistant (TA) Grade 1 (Junior). He moved up through all research grades, reaching Chief Research Scientist (CRS) in 1992. He was proud of being the only CSIRO employee to have ever achieved the feat of moving up from TA to CRS. He undertook a part-time degree in Food Technology at the University of New South Wales (UNSW), then continued with an MSc qualifying course at UNSW, and then a part-time MSc on “Microbiological Problems in Prune Preservation”. In these early years he worked on the preservation of dried fruit, which led into his life-long interest in mycological aspects of food spoilage. A full-time PhD at the University of California, Davis, studying yeast taxonomy, was then followed by a post-doctoral year at the USDA Northern Regional Research Center, Peoria, Illinois, where he first learnt *Penicillium* taxonomy. These years set up the two main streams to John’s science: John the taxonomist, the monographer of *Penicllium* (Pitt 1980), and John the food mycologist.

John the food mycologist sought to understand the relationships between fungi and food spoilage and the production of mycotoxins in the food supply, working on improving the methods and media used to isolate fungi from foods. He provided practical help and advice on mycological problems to the Australian food industry, from paddock to plate. He undertook projects with the Australian peanut industry, investigating ways to prevent the formation of aflatoxin in peanuts by using biocontrol strains of *Aspergillus* that could not produce aflatoxin. He worked with the Australian wheat industry on the quality of export wheat. He collaborated on projects in the Riverina region to understand and ameliorate the threat of ochratoxin contamination of Australian wine and sultanas. In South-East Asia, he worked on ACIAR funded projects on dried fish, and a wide range of durable commodities, to assess quality and potential mycotoxin problems.

John was there for the Australian food processing industry, providing advice and assistance in managing spoilage problems cause by preservative resistant yeasts, heat resistant moulds, xerophilic fungi, *Penicillium,* and *Aspergillus* in products as diverse as beverages, fruit purees, syrups, confectionery, cakes, bread, flour, tomato paste. For all these projects, and many others, John employed and trained young graduate scientists, and PhD students, many of whom went on to have very successful careers elsewhere in microbiology, food science and food mycology in Australia and abroad.

John built up a significant network of colleagues within Australia and overseas and was highly respected. His expertise in mycotoxins and foodborne fungi resulted in invitations to speak at conferences, giving many keynote and plenary addresses. He was valued for his productive contributions on many high-level international committees and working parties, including ICMSF, ICFM, WHO, and FAO. In 2003, John Pitt was awarded the Centenary Medal, Commonwealth of Australia, for “Services to Food Science and Technology”.

John was always very generous with his knowledge and loved teaching. With his North and South American colleagues, he organised and taught many workshops on *Penicillium* and *Aspergillus* identification, food mycology methods, and identification of foodborne fungi. This enthusiasm to pass on knowledge has been the driving force behind the 645-page 4th edition of *Fungi and Food Spoilage*, published in September 2022 (Pitt and Hocking 2022); it is so sad that he did not see the finished product.

On a personal level, John was our colleague, mentor, and friend, always available to provide advice and encouragement. We shared many fine meals and glasses of good Australian wine and enjoyed countless hours of conversation with him. He provided invaluable support to our careers and to the careers of so many others. He was also a demon on the dance floor and a master behind the barbeque. He will be greatly missed.

**Ailsa Hocking, Dee Carter & Wieland Meyer**

(wieland.meyer@sydney.edu.au)

## BOOK NEWS

### The Lives of Fungi: a natural history of our planet’s decomposers. By Britt A. Bunyard. 2022. Princeton: Princeton University Press. pp. 288. ISBN 978-0-691-22,984-3 (hbk), 978-0-691-23,035-1 (ebk). Price: £ 25 (hbk)

(Fig. 21).

**Fig. 21:**
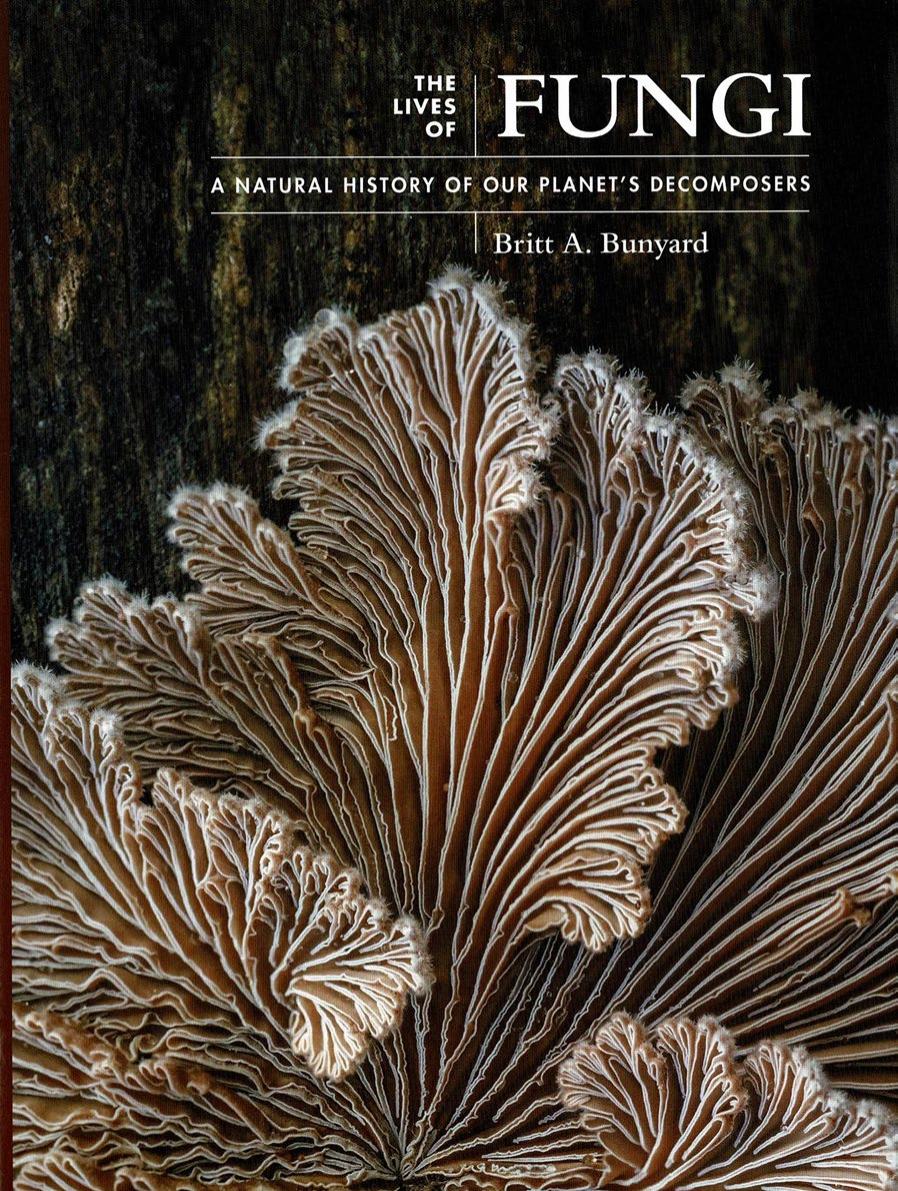
The lives of fungi (2022)

Although perhaps not so well-known internationally, the name Britt Bunyard will be familiar to North American field-oriented mycologists. He is a mycological researcher who has turned to energetically promoting mycology, and is now Executive Director of the now massive annual Telluride Mushroom Festival in Colorado, something that started in 1981 and is now an extraordinarily popular and diverse event attracting huge crowds (see e.g. https://www.youtube.com/watch?v=_VdENyhwVCA), celebrating all things mycological and today also includes academics amongst its speakers. He is also the Founder and Editor-in-Chief of the quarterly *Fungi* magazine, which he launched in 2008, and author of both specialist monographs and amateur-oriented books, include a well-received beginner’s guide (Bunyard and Lynch 2020).

This lavishly illustrated new book is simply a delight, and introduces the fungi not by any systematic arrangement, but refreshingly by grouping them according to ecological and functional roles: saprobes and parasites; pathogens, pandemics, and scourges; mutualistic symbionts; and fungi and humans. In each category, after providing an overview including information on aspects such as life-cycles, in many cases with lucid diagrams, there are detailed treatments of exemplar species. The selected species range from the microscopic (e.g. *Cryptococcus gattii*) and the Hat Thrower (*Pilobolus crystallinus*) through Horsehair Lichens (*Bryoria tortuosa* and *B. fremontii*), and Beech Orange Fungus (*Cyttaria gunnii*) to the Titan Mushroom (*Termitomyces titanicus*) and Stonemaker Fungus (*Laccocephalum mylittae*). Each appears as a double-page spread; the left page with text, frequently also explanatory diagrams, and always a world map of the known distribution; and the right with an especially eye-catching full-page colour photograph.

The fungi and humans section emphasizes the changing Earth climate and the carbon crisis, threats posed, problems in homes and gardens, beneficial fungi, and unwelcome fungi. The final section, fungi and the future, examines fungi that heal and feed, in food and industry, that kill, magic mushrooms, and extreme fungi. I found the section on magic mushrooms especially interesting, with much on the history of their re-discovery in the late 1950s and increase in awareness outside Central and South America. That section includes an undated photograph of Laura Huxley, widow of writer Aldous Huxley, which I had not seen before and intrigued me; she is with Timothy Leary who was much involved in experimentation with psilocybin and other hallucinogenic compounds in the 1960s. This is not expanded on in the text, but while Aldous is well-known to have also experimented and reported on his experiences, it is not often appreciated that Laura was the one who encouraged such personal experimentation.

Understandably, and perhaps inevitably, there is something of a North American bias, but not an overwhelming one. This is especially so in the list of “useful resources” of books and, organizations, and websites which contains some rather dated texts and omits some sources that would be very valuable for field mycologists in Europe.

This is a really enjoyable text, and I cannot better the comment on the back cover that it combines “engaging and accessible text with beautiful images” and “lays out all the essential facts about fungi for the mycologically curious”. It will do much to promote the awareness of fungi, and merits a wide circulation throughout the world and not only in North America. It is an achievement of which the author should be extremely proud.

### The Magic of Mushrooms: fungi in folklore, superstition, and traditional medicine. By Sandra Lawrence. 2022. London: Welbeck (in association with the Royal Botanic Gardens Kew). pp. 208. ISBN 978-1-78,739-906-8 (hbk). Price: £ 14.99 (hbk)

(Fig. 22).

**Fig. 22:**
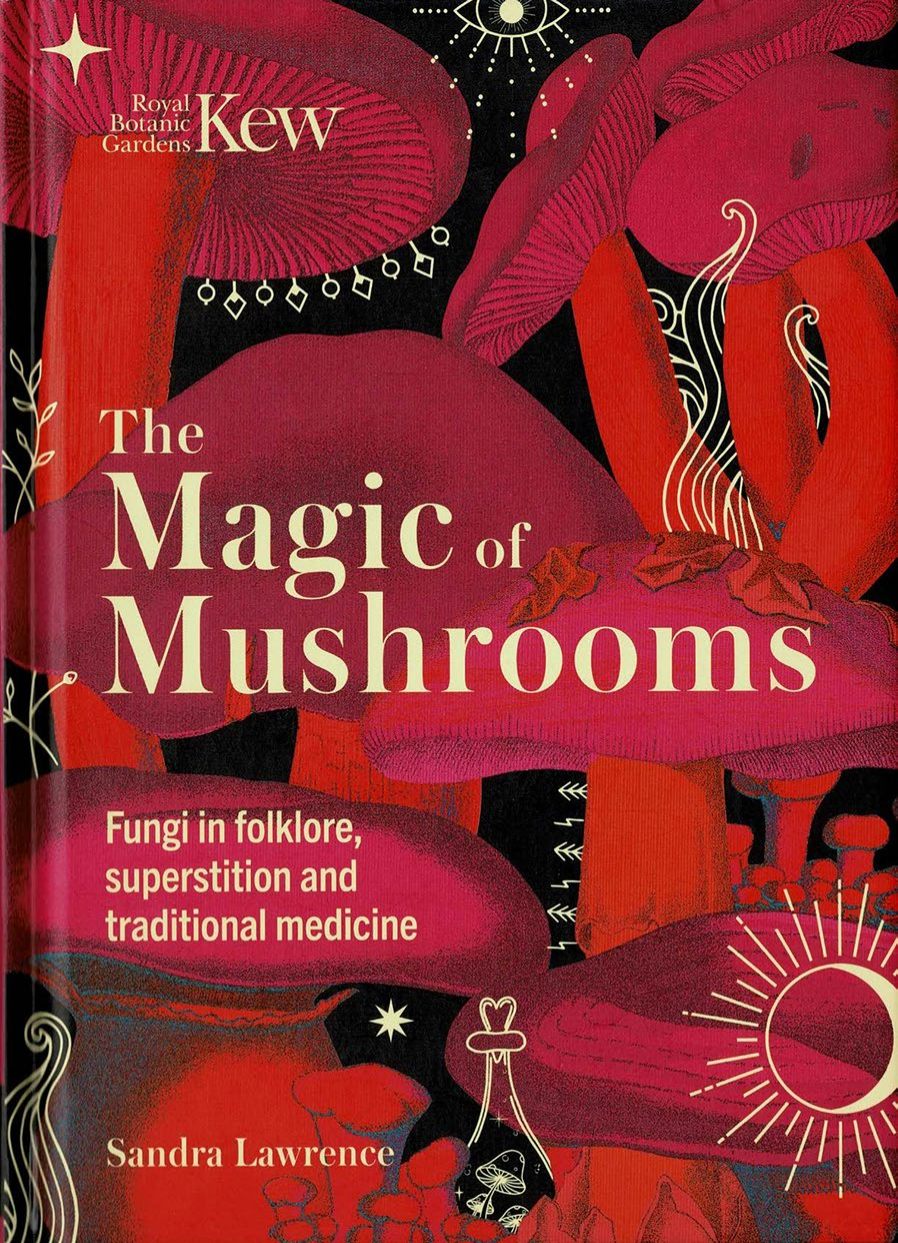
The magic of mushrooms (2022)

This book has quite a different approach from the *Lives of Fungi* in being focussed, as the subtitle makes clear, folklore, superstition, and traditional medicine. It asks the general reader to “immerse yourself in a bewitching world and discover the life-changing potential of fungi”. Sandra is an experienced writer and journalist, with an engaging style and this is about her 16th non-fiction book. It is clear from the text that she has really enjoyed this exploration into some of the darker aspects of mycology, and that “we are still fascinated, mystified and perhaps a little terrified by fungi”.

The chapter headings reflect not only the scope but her style: The human history of fungus; How mushrooms work; Great Minds; Fairy rings; Fungus in food; Fungus in art; Flying high; The cunning woman’s stillroom; The Dark Mirror, the grim side of fungus; and The future of fungus. She has carried out an extraordinary amount of research delving into historical literature and paintings, and every page is packed with facts, many of which will be unfamiliar to even the professional mycologist. An especially enjoyable feature is that the numerous illustrations are all reproduced in colour from paintings and classic historic treatises mostly from the eighteenth and nineteenth centuries but with some from the 16th and even earlier depictions. Mrs Hussey’s *Illustrations of British Mycology,* published from 1847 to 1855, is a particularly favoured and welcome source as many mycologists will never have seen an original of that splendid work. I noted only a single photograph – a Victorian one of a woman posing by a giant model “toadstool”.

I was pleased to see that she does not avoid the controversial and sometimes emotive debate over the role of hallucinogenic mushrooms may have had in early religion, reproducing a twelfth century fresco of Adam and Eve flanking a giant mushroom. Her accounts are self-contained, easily readable, and recounted in very manageable portions. Frequently just one or two pages on a particular theme are faced by a generally full-page illustration of the mushroom under consideration. This makes the whole ideal to dip into for just short periods of relaxation.

In preparing this text, Sandra has had the benefit of help from Rich Wright and Lee Davies on the Kew staff, and a majority of the images come from the Library and Archives of the Royal Botanic Gardens Kew. With its emphasis on folkore and A5 size, this book is a good companion to Kew’s large-format *Fungarium* (Scott & Gaya 2019) reviewed in *IMA Fungus* **11** (28): 28–29, 2020) and merits a wide readership.

### Larger Fungi in Eastern Tropical Africa: a field guide. By Tuomo Niemelä, Marja Härkönen, and Graham Pierce. 2021. [Norrlinia Vol. 36.] Helsinki: Finnish Museum of Natural History. pp. 336. ISBN 978-951-51-7641-7 (pbk). Price: 30 €.

(Fig. 23).

**Fig. 23:**
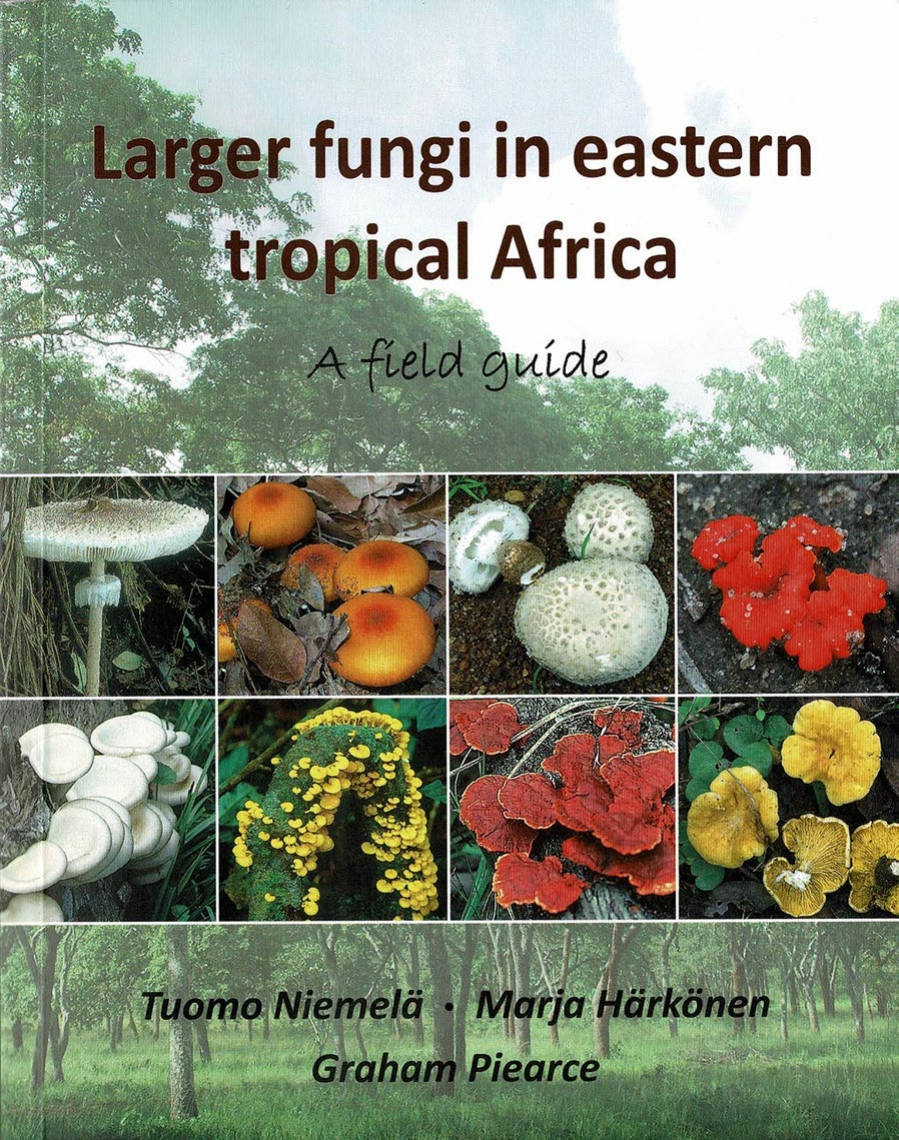
Larger fungi in Eastern Tropical Africa (2021)

This book builds on earlier works by the same group of Finnish mycologists which have focussed on Tanzania and Zambia and had more restricted circulations as they were tailored for use as textbooks by forestry scholars. This new work, also embraces Ethiopia, Kenya, and Mozambique and is aimed at a more general audience. It is based primarily on collections made by the authors in travels in the region, and includes a map of routes taken (p. 299). It is primarily an identification guide for macromycetes with 173 pages not unsurprisingly devoted to gilled mushrooms and boletes, and then smaller amounts to polypores, gastroids, etc.

Over 250 species are treated in detail, and an especial feature is the superb 470 or so colour photographs. Of particular importance is the citation of the collection details, with localities and indications of where the vouchers are preserved. This is a most welcome practice that merits widespread adoption as best-practice, and amongst these are illustrations of 33 type collections. For each species there is description, including a small-print paragraph of microscopic details of the spores or other minute features, and a paragraph of Notes outlining the ecology, in some cases edibility or toxicity, and with notes on identification. The authors are also frank when they are uncertain over identifications and indicate where these are tentative, as is in case of *Armillaria fuscipes.*

There is, however, much more here than a straight identification manual. There are descriptions of key habitats, Miombo woodlands, *Acacia* savannas, Mountain forests, and Tree plantations, noting the issue of introduced fungi such as *Amanita* species and *Suillus granulatus*. The value of fungi to people and ecosystems is explained, and by using a questionnaire information on how local peoples viewed and used macromycetes was gleaned from 35 different tribes in Tanzania and 13 in Zambia. There are nine pages of recipes, mainly gathered by Marja Härkönen from interviewees, with in some cases preparation steps illustrated as well as the final dishes. The book closes with a glossary which includes some most helpful line drawings, a list of further reading listing key works from the region, and a species index.

The authors have produced a work which will be of immense value and a stimulus to upcoming mycologists in a region much of which remains relatively unexplored even for macromycetes. One can only concur that “There is still so much to find in African mycology” (p. 7) and hope that this fine book will help encourage more local people to explore and document their funga.

### Fungal Wealth of the Western Ghats: glimpses of fungal diversity. Vol. 1. By Kiran Ranadive, Ivan Zmitrovich, Alina Alexandiva, and Raman Kulkarni. 2022. Puna: Kiran Ramchandra Ranadive. pp. 304. ISBN 978-3-5607-082-0 (hbk). Price: ₹ 2800/-

(Fig. 24).

**Fig. 24:**
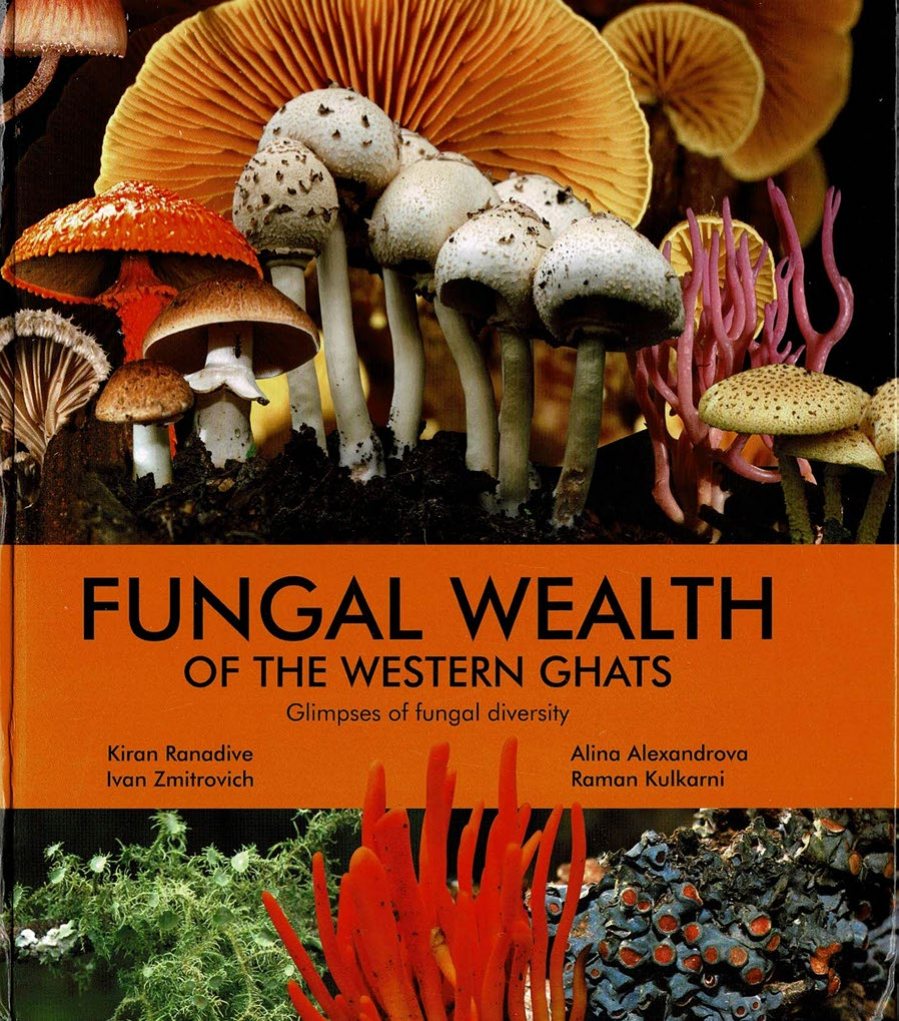
Fungal wealth of the Western Ghats (2022)

India has a strong mycological tradition, and a vigorous mycological society, but attractive outreach publications are few and far between. Kiran Ranadive took the initiative to produce this really stunning guide because, as he states in the Preface, he found from popular talks he had given that there was much interest amongst students and lay people to learn about the fungal life-style. Indeed, it was being asked if there was any book to help them that inspired him to launch into this project. In order to make this venture a reality, he was able to recruit the assistance of Russian mycologists Ivan Zmitrovich and Alina Alexandiva, and also the experienced Indian wildlife photographer Raman Kulkarni and to whom almost all the photographs are credited.

The Western Ghats in south-west India is one of the world’s acknowledged biodiversity hot-spots, with not only over 1700 endemic plants species, but is an area from which numerous fungi, including lichen-forming species, continue to be described as new. The first chapters of the book discuss the various fungal life-styles and habitats of lichen-forming as well as other fungi, life-cycles, classification, uses, and ecological roles. The classification system presented is sadly one almost 50 years old and consequently very dated. The system even recognizes *Deuteromycotina* as a subphylum on a par with *Ascomycotina,* notwithstanding the one-fungus = one-name concept having been explained ten pages earlier.

The primary mission of the book is, however, to provide glimpses of the fungal wealth of the area. In what is planned to be the start of a series of volumes, 137 species from across the fungal spectrum are featured. A double-page spread is devoted to each of the 120 non-lichenized species, and the 17 lichenized have a single page each. The photographs can only be described as stunning and are a real tribute to the expertise of the photographer. The accompanying text provides the scientific and English common names (but not local Indian names), family placement, colour, habitat, host specificity (if any), any special features, a description, and world distribution – but perhaps wisely no indication of edibility or toxicity. The book concludes with an extensive glossary, index, references which in addition to works cited include many on the fungi of the region, and web resources.

The whole is beautifully laid out and sure to capture the imagination of anyone wishing to start to explore fungi in the region, but it is also of wider importance as it includes species described from India of which colour images may not have previously been published. While the price is very reasonable for such a lavish book in western eyes, equating to about £ 28 or 34 US$, I do worry that this might hinder the circulation in the region it merits.

I can only congratulate the authors on the production of such a fine work, and trust that it will fulfil the start of their hope “that this book will definitely serve as a promising step towards conservation of the remaining Indian fungal biota by creating interest in the new generations”. As the series continues with future volumes, that will undoubtedly increase its importance, and I wish the authors all success in furthering this important initiative.

### Applied Mycology: entrepreneurship with fungi. Edited by Amritesh Chandra Shukla. 2022. Cham, Switzerland: Springer Nature. pp. xviii + 447. ISBN 978-3-030-90,648-1 (hbk), 978-3-030-90,649-8 (ebk). Price: £ 179.99 (hbk), £ 143.50 (ebk)

(Fig. 25).

**Fig. 25:**
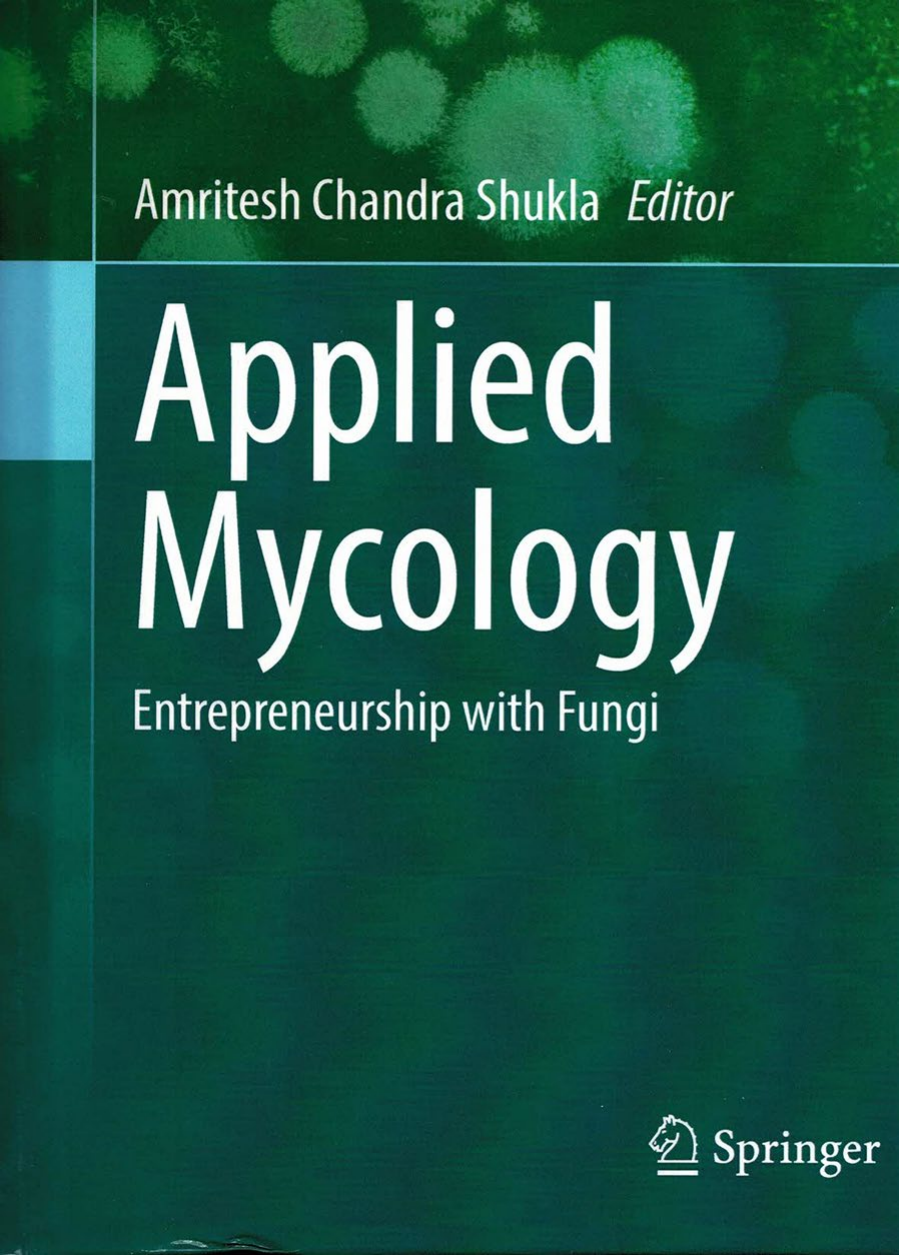
Applied mycology (2022)

Most mycologists will be aware that there are numerous opportunities for entrepreneurs to exploit attributes of fungi, but which never have the investment to take maximum advantage of their attributes. This volume, comprising 20 chapters, has 60 contributors, all but one from a university, and just eight from outside India. The range of possibilities discussed is too enormous to detail here, but is almost unimaginably diverse: agricultural productivity, baking, biodegradation, biofertilizers, biofungicides, bioherbicides, cosmetics, decontamination, enzymes, food, mycoremediation, nanoparticals, neutraceuticals, pharmaceuticals, etc. The editor has done well to bring such a diverse range of topics together, yet the coverage is not exhaustive. Areas not or hardly touched on include dyes, manufacture of construction and packing materials, pesticides, pigments, and mycoprotein production are missing.

Several chapters include flow diagrams of processes or steps to be followed, many in colour and generally very clear. The list of references at the end of every chapter is particularly impressive and will be of especial value for anyone wishing to explore a particular possibility further. The “elephant in the room”, however, is how to scale-up from a laboratory or experimental plot setting to a factory or whole field situation, secure intellectual property rights (e.g. patents), and still have a commercially viable product with a defined market and marketing plan. This process is something that would in most cases require convincing potential venture capitalists to invest in new start-up companies; such activity is outside the remit of many universities.

A particular value of this work, however, will be that it can be shown to anyone who doubts about the potential of fungi to contribute to human well-being and economic development, especially as there does not appear to have been such a wide-ranging overview prepared in the last decade (even in volumes of Springer’s *The Mycota*).

### Lichens: toward a minimal resistance. By Vincent Zonca. 2022 [“2023”]. Cambridge, UK: Polity Press. pp. xvii + 261. ISBN 978-1-5095-5344-0 (hbk), 978-1-5095-5345-7 (pbk), 978-1-5095-5346-4 (ebk). Price: £ 55 (hbk), £ 21.90 (pbk, ebk)

(Fig. 26).

**Fig. 26:**
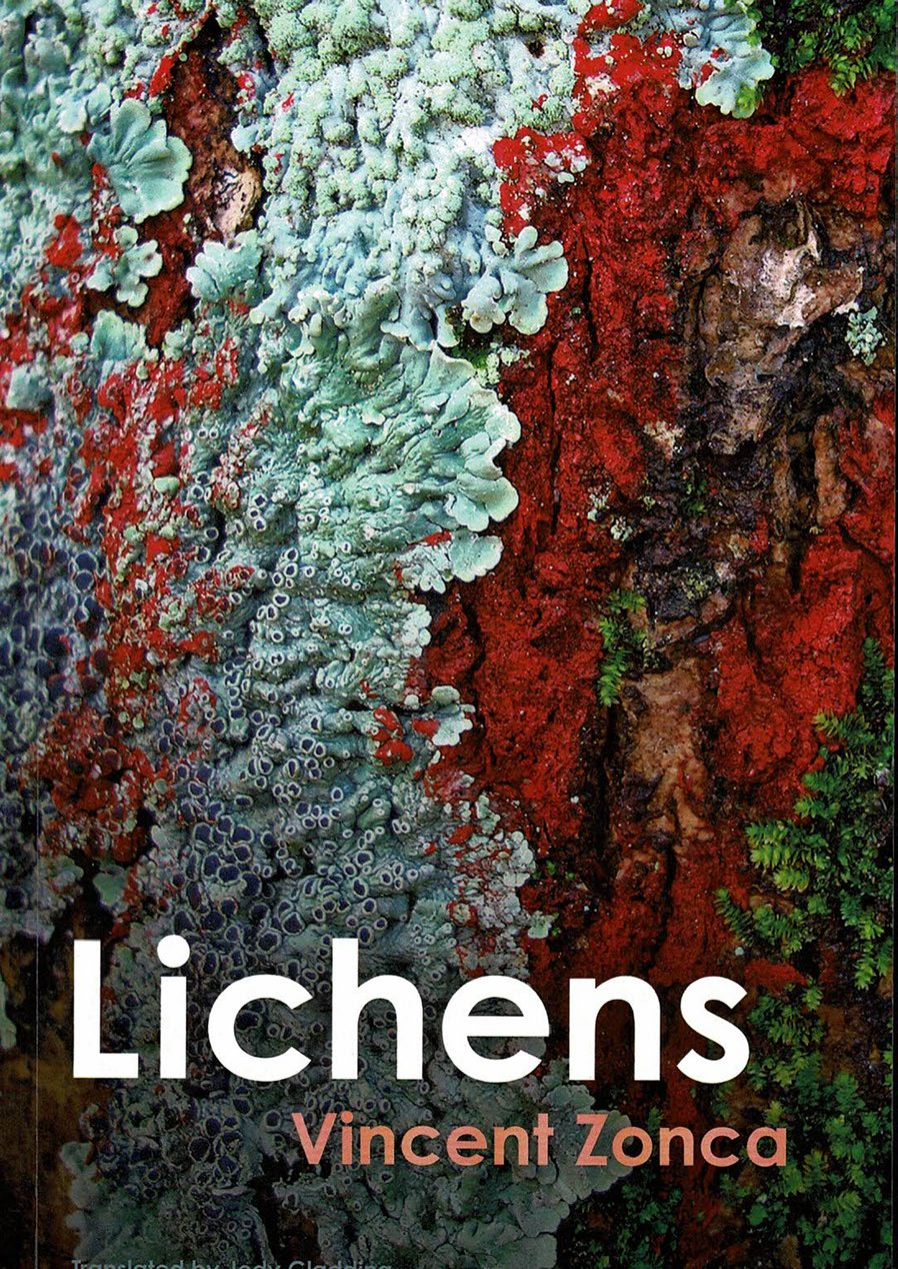
Lichens (2022)

This is a most unusual book, originally published in French in 2021, and now translated into English by Jody Gladding. The author, a researcher and writer who lives in Brazil, became fascinated by lichens and then spent several years investigating them around the world. The result is a work that is at the interface of art and science, aimed at a popular audience, who, after reading it asserts the back cover “will never see lichens, or the world, in the same way again”. I can only concur.

It ranges through the history of “lichen”, elucidation of their nature, customs and beliefs, erotics, representation, ecopoetics, sentinels and forewarnings, resistance, poethics”, microhabitats, politics, chimeras and vampires, cohabitation, etc. Each section has detailed endnotes and references to his impressively wide range of sources, from the classical to research papers published in the last few years. The style and approach recalls that of Pierre Gascar’s *Le Présage* (Gascar 1972), published only in French, and which Zonka acknowledges and cites at various points.

This is a book to enjoy and reflect on, especially if you are already fascinated by lichens. It will be sure to introduce you, as it did me, to new ways of looking at lichens that you had never previously thought of. A delightful work for a long flight or to dip into at the end of a busy day.

## NOTICES

1. *MycoNews* is compiled by David L. Hawksworth as Editor-in-Chief, and to whom all material for consideration for inclusion in *MycoNews* should be sent directly by e-mail.
2. Books for possible coverage in the Book News section should be mailed to David L. Hawksworth at Milford House, 10 The Mead, Ashtead, Surrey KT21 2LZ, UK; works issued only as e-books are not normally included, but reviews prepared by others will also be considered if sent to him.
3. Reports of new genome sequences intended for inclusion in the *Fungal Genomes* compilation should be sent directly to Senior Editor Brenda Wingfield as e-mail attachments and not submitted through Editorial Manager.
4. All unsigned items in *MycoNews* can be attributed to the compiler, David L. Hawksworth.
